# New perspectives in regenerative medicine and surgery: the bioactive composite therapies (BACTs)

**DOI:** 10.1007/s00238-021-01874-6

**Published:** 2021-10-29

**Authors:** Michele L. Zocchi, Federico Facchin, Andrea Pagani, Claudia Bonino, Andrea Sbarbati, Giamaica Conti, Vincenzo Vindigni, Franco Bassetto

**Affiliations:** 1 Plastic and Reconstructive Surgery Unit, University of Padua, Padua, Italy; 2 Remix Institute for Regenerative Surgery, Turin, Italy; 3 Department of Plastic and Hand Surgery, Technical University of Munich, Munich, Germany; 4 Department of Rheumatology and Immune Diseases, Humanitas Gradenigo Hospital, Turin, Italy; 5 Institute of Human Anatomy, University of Verona, Verona, Italy

**Keywords:** Minimal grade manipulation (MGM), Programmed cell death (PCD), Bioactive composite mixtures (BACMs), Adipose-derived stem cells (ADSCs), Stromal vascular fraction (SVF), Cellular components (CCs), Blood components (BCs), Platelet-rich fibrin (PRF), Amino acids (AA), Vitamins, Reduced glutathione (GSH), REMIX, Cytokine modulation, Donor site preparation, Delayed harvesting, Muse cells, Photobiostimulation, ROS antagonism

## Abstract

Regenerative medicine and surgery is a rapidly expanding branch of translational research in tissue engineering, cellular and molecular biology.

To date, the methods to improve cell intake, survival, and isolation need to comply with a complex and still unclear regulatory frame, becoming everyday more restrictive and often limiting the effectiveness and outcome of the therapeutic choices. Thus, the authors developed a novel 360° regenerative strategy based on the synergic action of several new components called the bioactive composite therapies (BACTs) to improve grafted cells intake, and survival in total compliance with the legal and ethical limits of the current regulatory frame.

The rationale at the origin of this new technology is based on the evidence that cells need supportive substrate to survive in vitro and this observation, applying the concept of translational medicine, is true also in vivo. Bioactive composite mixtures (BACMs) are tailor-made bioactive mixtures containing several bioactive components that support cells’ survival and induce a regenerative response in vivo by stimulating the recipient site to act as an in situ real bioreactor. Many different tissues have been used in the past for the isolation of cells, molecules, and growth factors, but the adipose tissue and its stromal vascular fraction (SVF) remains the most valuable, abundant, safe, and reliable source of regenerative components and particularly of adipose-derived stems cells (ADSCs). The role of plastic surgeons as the historical experts in all the most advanced techniques for harvesting, manipulating, and grafting adipose tissue is fundamental in this constant process of expansion of regenerative procedures. In this article, we analyze the main causes of cell death and the strategies for preventing it, and we present all the technical steps for preparing the main components of BACMs and the different mixing modalities to obtain the most efficient regenerative action on different clinical and pathological conditions. The second section of this work is dedicated to the logical and sequential evolution from simple bioactive composite grafts (BACGs) that distinguished our initial approach to regenerative medicine, to BACTs where many other fundamental technical steps are analyzed and integrated for supporting and enhancing the most efficient regenerative activity. Level of Evidence: Not gradable

## Introduction

Regenerative medicine is today considered the newest and most promising global health pillar based on the clinical application of cell therapy by promoting and stimulating the body’s own repair mechanisms [1]. Mesenchymal stem cells (MSCs), first described by A. Caplan in 1989, have shown a unique potential in repairing damaged tissues and organs and active regeneration whenever compromised by the most different etiology and reasons, ranging from acute causes and trauma to chronic and degenerative conditions. Cell therapy exhibits unique advantages, such as its ability of restoring tissue function, together with high viability and low morbidity [2–4].

**Fig. 1:**
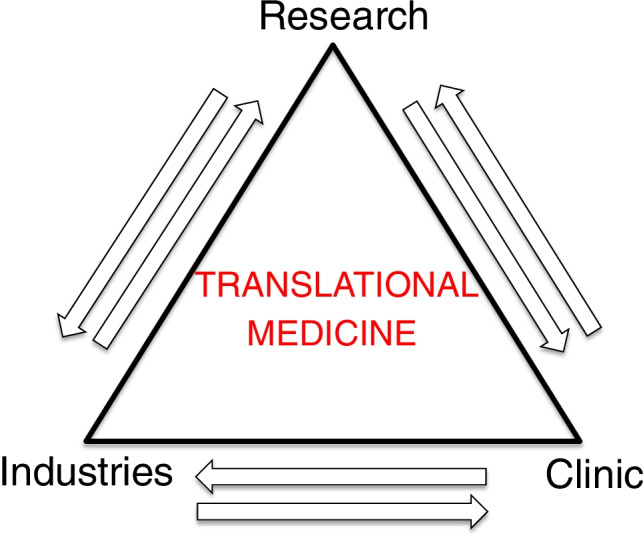
The fundamental principle of translational medicine is to establish and maintain a close correlation between research, industry, and clinical applications. Its golden rule is “bench to bedside”

The translational approach of applying in vitro studies to clinical practice and needs recently allowed great advances in this challenging field. The third important side of the golden translational triangle, represented by industry, showed an increasing interest in actively supporting the technical aspects of this blooming field of research for improving advanced regenerative strategies for the most challenging clinical applications [2, 5, 6] (Fig. 1).

When tissues get damaged, the most common body’s repairing response is represented by the scarring activity, which, producing nonfunctional tissue only, interferes and limits the scope of regeneration. A real and effective regenerative process should ensure complete functional and morphologic restoration and this goal can be achieved only through functional cell division and concomitant self-renewal and differentiation of mesenchymal stem cells.

Medicinal signaling cells (MSCs), previously known as mesenchymal stem cells, are multipotent stromal cells originating from the three mesenchymal layers (exothermic, mesodermic, and endodermic) able to differentiate into many cellular lines such as osteoblasts, chondrocytes, adipocytes, endothelial, muscular, and nervous cells [7]. MSCs are strictly in contact with the basement membrane and with endothelial cells of the microvascular net. During a regenerative process, MSCs become functional and establish a local regenerative microenvironment called “niche” for ensuring metabolic and chemotactic exchanges. Specific markers such as NG-2b and CD146 are expressed both on the surface of isolated MSCs and on pericytes, a cell population within the microvascular net [8]. This allowed A. Caplan to equalize MSCs with pericytes, considering them as in vivo site-regulated “drugstores” [9].

Among different sources of MSCs, adipose tissue (AT) remains one of the most promising, valuable, and reliable sources of regenerative elements as adipose-derived stem cells (ADSCs) [10, 11].

Active tissue healing and extracellular matrix (ECM) deposition are even promoted by local synergic support of cells, molecules, and GFs. In fact, the secretome derived from MSCs may contribute to the regeneration of the tissue microenvironment in damaged or injured areas. The MSCs have been shown to secrete bioactive factors that affect immune systems’ cells and functions such as inhibition of apoptosis, enhancement of cellular migration, promotion of angiogenesis, and increase of the rate of proliferation of stem or progenitor cells present in the tissue in a process called “trophic activity” [2].

**Fig. 2:**
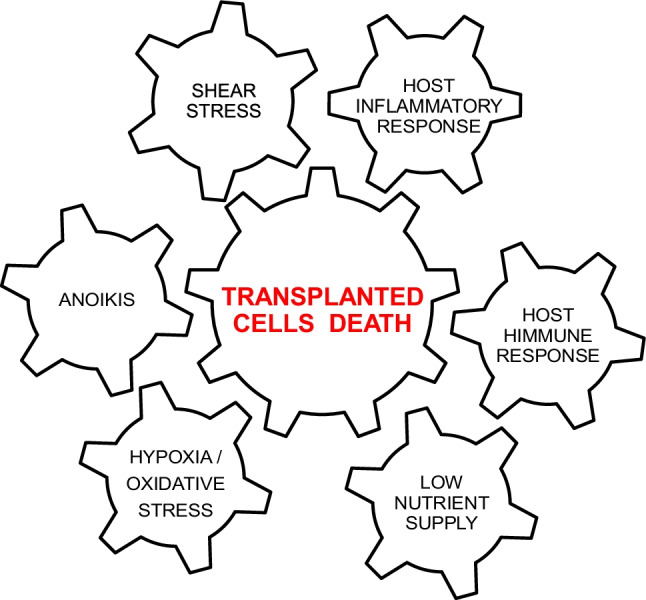
Major causes of transplanted cell death

Nonetheless, from the pathophysiological point of view, there are different elements deeply interfering with transplanted cell survival and local homeostasis and, consequentially, induce transplanted cell death (TCD) within the recipient site: host inflammatory response, host immune response, shear, and mechanic stress, the local activity of reactive oxygen species (ROS), hypoxia, and low nutrient supply are the most relevant anoxia leading to programmed cell deaths (PCD) consequent to anoikis and apoptosis [12]. Whereas mechanical stimulation and crushing can be at the origin of shear stress, the inflammatory host and immune response are associated with local and systemic cytokines activation; low nutrient supply and hypoxia are key in several metabolic stress processes while PCD for anoikis, due to ECM separation from cellular components, and apoptosis seems to be sustained by abnormal activity of death-associated protein 3 (DAP3) (Fig. 2, Table 1A).

**Table 1:** (A) Shear stress, inflammation, and immune response are responsible for mechanical crushing and cytokines activation. Conversely, low nutrient supply and hypoxia are the key for different metabolic processes and anoikis is involved in ECM separation. (B) The strategies for transplanted cell survival are represented by the action of growth factors, tissue engineering principles, tissue conditioning, ROS protection, and complement inactivation. Cell expansion, cultivation, and stimulation of proapoptotic pathways are not allowed in the clinical practice

A	
**Major causes of transplanted cell death**	**Mechanism of cell damage**
Shear stress	Mechanical stress
Host inflammatory response	Cytokine activation
Host immune response	Cytokine activation
Low nutrient supply	Metabolic stress
Hypoxia/oxidative stress	Metabolic stress
Anoikis	Separation from ECM
B	
**Major strategies for supporting transplanted cell survival**	**Operative action**
Tissue/cell conditioning	Graft/donor site stimulation
Cytokine modulation	Probiotic redirecting
ROS protection	Antioxidant enzymes
Anoxia/hypoxia protection	O2 implementation
Complement inactivation	Plasmin activation
Advanced tissue engineering	Expansion/cultivation
Pro-survival gene transfer	Proapoptotic pathway

All these elements, working in a deleterious synergism, can consistently jeopardize transplanted cells integration to the host recipient site by interfering with niche homeostasis.

For these reasons, the authors, for many years now, dedicated consistent efforts to analyze and develop new strategies aimed to improve transplanted cell survival (TCS). Tissue engineering, gene transfer, preconditioning procedures, ROS protection, complement inactivation, cytokines modulation, and probiotic redirecting showed very promising potential in favoring ADSCs precursors intake, survival, and differentiations [13].

**Fig. 3:**
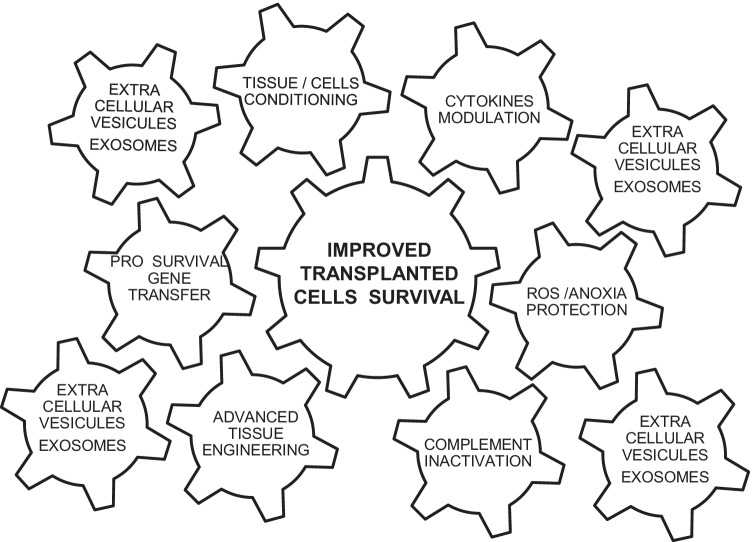
Strategies to improve transplanted cell survival

Pro-survival factors are mainly supported and mediated by extracellular membranous vesicles, better known as exosomes, containing mRNAs and signal molecules, able to mediate intercellular communication by transporting proteins or nucleic acids into target cells thus altering the behaviors of recipient cells. The combination of all these pro-survival factors sustains, through positive synergic interaction, niche homeostasis, and allows the transplanted therapy survival inside of the host recipient site (Table 1B, Fig. 3).

However, not all those procedures are, at the present time, legally allowed for human treatment in most countries, and beside very few exceptions; the current status of the regulatory frame of regenerative technologies is fairly standard and more or less patterned on the US FDA guidelines [14]. The possibility of using cell derivatives obtained with in vitro expansion and cultivation of MSCs for human implantation is still very limited almost everywhere by the existing legal issues [15].

In the majority of developed countries, only cells or nonstructural tissues-based therapies that do not cause relevant alteration of the biological characteristics of cells or tissues and preserving their original properties and functions, such as decantation, filtration, centrifugation, and mechanical disruption without relevant alteration are allowed in the clinical practice. These methods are included in the definition of minimal-grade manipulation (MGM), by which tissues are harvested, treated, and reimplanted during the same surgical session and inside the same operating room (Zocchi et al. [16]).

On the contrary, high-grade manipulation (HGM) techniques, including cell characterization, expansion, cultivation, and all the other complementary laboratory techniques routinely used for cell manipulation, cannot be used. Few are today, the exceptions where the use of cellular derivatives obtained through HGM and heterologous and allogeneic cells transplantation is allowed for daily practice [17]. Therefore, simple and well-consolidated laboratory procedures, such as enzymatic digestion of the adipose tissue with collagenase (0.075% dilution for 30 min at 37°), centrifugation, incubation, expansion in a Dulbecco’s modified eagle medium (DMEM) with 10% fetal bovine serum and antibiotics and subsequent cultivation toward specific lines of cellularity, cannot be used as a routine choice for MSC transplantation and therapies [16].

Nonetheless, carefully analyzing the current ethical, legal, and the regulatory frame is merging the evidence that other technical steps, such as tissue and cell conditioning procedures, both in donor and in the recipient site, the use of autologous GFs and exosomes, cytokine modulation and redirecting, complement inactivation, and ROS protection are permitted and can be therefore integrated with due care and precautions in regenerative protocols. Conversely, advanced tissue engineering procedures such as expansion or cultivation and pro-survival gene transfer processes are still not allowed.

### Bioactive composite therapies

#### The rationale behind the strategy—the translation of in vitro procedures to in vivo practice

The need to find more efficient strategies to support cell therapies made the authors focus on the basic principles of translational medicine. Thanks to a constant exchange of knowledge with major world leaders in this field and in parallel with the evolution from tissue engineering to advanced regenerative medicine, authors widened their approach integrating to the use of the bioactive composite grafts (BACGs) already described in previous articles [16] and the synergic action of the newest tools for improving grafted cell intake and survival. This novel 360° regenerative strategy is called bioactive composite therapies (BACTs) according to the precious suggestion of Prof. Fu-Chan Wei [18].

Recognizing all the above-mentioned factors able to improve cell survival and proliferation made, the authors aimed in reproducing the in vitro cell plate conditions to a host recipient site in vivo to improve grafted cell homeostasis, intake, and survival still respecting the existing regulatory frame limits.

The main concept behind this new line of research is that for obtaining the most efficient regenerative activity, tuned for every clinical need and for every anatomic area, it is mandatory to use the synergic action of several bioactive components. Instead of injecting a “single-component graft” alone (i.e., ADMCs), a multicomponent bioactive mixture called BACMs with enhanced regenerative activity, can be used to support transplanted cells kick-starting different regenerative processes and inducing the recipient site to act as a real bioreactor. Different regenerative mixtures can be conceived and tailored for each tissue and anatomic area and different clinical needs.

Nonetheless, to facilitate and support the applicability of the procedure, all the technical steps for harvesting, isolating, and concentrating the different regenerative bioactive components should be affordable to the patient and to the surgeon.

#### Composition of the bioactive composite mixtures

BACMs are mainly composed of two basic bioactive components:

1. Cellular components (CCs) isolated and extracted from a freshly adipose-derived stromal vascular fraction (SVF), either tissutal SVF with its ECM fraction or pure and concentrated cellular SVF with the sole cellular components very rich in ASC precursors;
2. Blood components (BCs), such as platelet-rich fibrin (PRF) very rich in specific and a specific GFs.

These two components alone represent, depending on the clinical needs, between 70 and 90% of their total volume. However, other important components should be added in minor proportions for completing the BACMs regenerative potential:

1. Type 1 bio-catalyzes, such as amino acids (AA), vitamins, and reduced glutathione (GSH);
2. Type 2 bio-catalyzes, such as specific morphoproteins;
3. Carriers such as HA, polysaccharides, and ECM.

These additional components could be crucial for redirecting, supporting, and enhancing the outcome of the two main bioactive components and should be added in different proportions and concentrations to the BACTs and grafted to the host recipient site. The proportion between SVF, BCs, and the other components depends on the clinical situation, on the therapeutic needs and on the recipient site’s anatomy and volume. Specific protocols have been established for adapting the bioactive preparation (Table 2).

**Table 2:** The components of the bioactive composite mixtures: adipose-derived stromal vascular fraction, blood components, bio catalyzers, and carriers

Basic components of the bioactive composite mixtures		
Adipose tissue derivates	SVF	MSCs and ECM
Blood components	PRGF, PL, PRF	Growth factors
Bio-catalyzer type 1	AA, vitamins, GSH	Cell intake and growth
Bio-catalyzer type 2	Morphoproteins	Linear differentiation
Carriers	HA, PCA, ECM	Facilitate implantation

The CCs and BCs are the key components of the BACTs and usually mixed in a ratio of 5:1 (e.g., for every 10 cc of volume of the cellular components, 8 cc are represented by ADSVF and 2 cc by PRF). The aim of BACTs technique is to increase the regenerative action by supporting cell intake, differentiation, and survival.

### Cellular component: stromal vascular fraction and ADSC precursors

Before entering into the technical details related to composition and preparation of BACMs and in order to avoid any confusion, the author would like to stress an important preliminary concept: the term mesenchymal stem cells (MSCs) and adipose-derived stem cells (ADSCs) too often are erroneously used by nonqualified professionals mainly for commercial purposes. Those acronyms should be used to identify only and solely those cultured cells after expansion, isolation, and cultivation. All the other cellular or tissue fractions obtained either with enzymatic digestion or with mechanical disruption of AT, without undergoing any of the abovementioned laboratory processes, should be instead identified as "freshly insulated SVF" containing a small fraction of MSC precursors and pericytes.

**Fig. 4:**
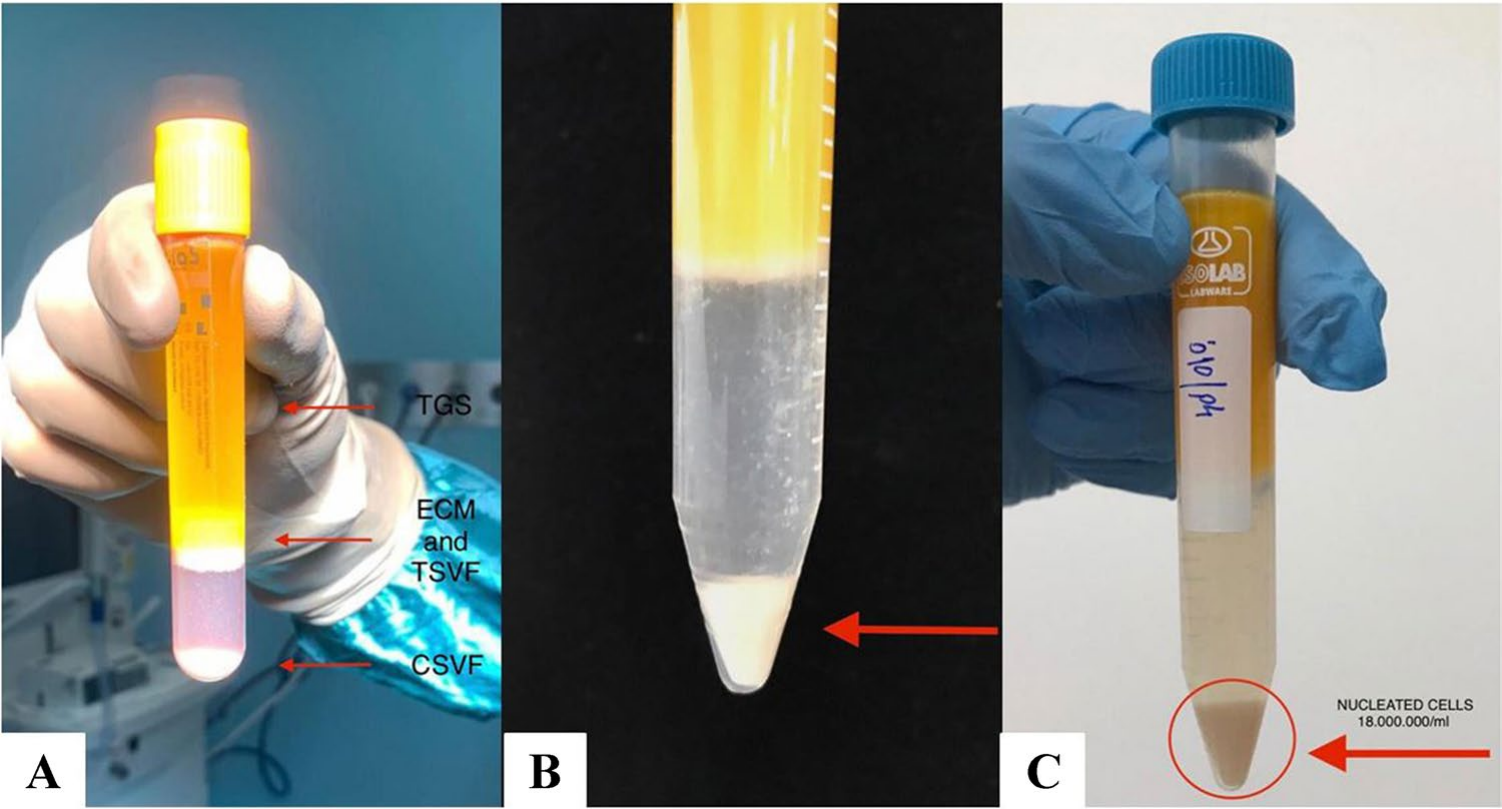
**A** **B** and **C** The stromal vascular fraction is a tissue-cellular mixture composed by a very heterogeneous pool of cells such as erythrocytes, lymphocytes, fibroblasts, monocytes, macrophages, endothelial cells, pericytes, and the extracellular matrix (ECM) is containing collagen, laminine, elastine, and other components. **C **Reports the cellular SVF that can be obtained with the most recent microlyzation procedures

The SVF is a heterogeneous cellular mix composed of ECM and different key cells types, including erythrocytes, lymphocytes, T regulatory cells, fibroblasts, monocytes, M2 macrophages, pericytes, preadipocytes, endothelial cells, and ADSC precursors (Fig. 4).

It is possible to identify two different fractions of SVF: the tissue SVF (TSVF) containing also the ECM component and the cellular SVF (CSVF) mostly composed by the nucleated cellular pool. This heterogeneous cell population must undergo a multiparameter flow cytometric assay to identify cells and relative sequences.

Besides the AT, other valuable sources of MSCs are bone marrow, blood, lungs, teeth’s dental pulp, and different fetal components such as placenta, amniotic fluid and membrane, and the umbilical cord. The previous research on MSCs was mainly focused on bone marrow mesenchymal stem cells (BMSCs). However, the discomfort and frequent pain associated with the invasive procedure for harvesting bone marrow and the limited number of MSCs that can be extracted from this source always represented an important technical limit for immediate grafting without cell cultivation and expansion (HGM). It has progressively lost popularity with the sole exception of the orthopedic surgery field, where it can still find some supporters.

Conversely, the adipose tissue can be easily isolated from multiple body areas with minimally invasive procedures. Nowadays, the AT is certainly the safest, abundant, and accessible source of MSCs with significant proliferative and multi-lineage differentiation potential toward osteogenic, chondrogenic, and adipogenic cell lines.

This observation underlines once again the fundamental role of plastic surgeons in the field of regenerative surgery as the historical experts in the most advanced and safe techniques for harvesting, manipulating, and grafting AT.

In fact, the number of MSC precursors contained in the AT-derived CSVF is significantly larger than in the bone marrow (up to 1000 times more) from 10 cc of decanted AT, with the existing technologies, it is now possible to insulate nearly 1 cc of SVF, containing up to 18,000,000 of nucleated cells. However, it is important to consider that only 5 to 7% of this cell pool, in the best scenario, is represented by MSC progenitors, which need to be isolated [19, 20].

## Methods of isolation

All the known techniques for adipose tissue manipulation require multiple technical steps: patient infiltration, AT harvesting, sample processing, isolation phase, enrichment, and eventually, injection in the recipient site. If these steps are realized following different technical protocols and devices, the final results will be unavoidably different for types of cells, number, vitality, viability, and characterization. The choice of the ideal method is therefore strictly dependent on the final tissue or cellular sample required and on its clinical application. Consistent differences exist between manipulated and nonmanipulated fat (i.e., mechanical-manipulation allows the isolation of fat samples with a way higher number of adipocytes and ADSC precursors and even a higher ADSCs-to-adipocyte ratio per volume if compared to nonmanipulated fat [21–26]). Centrifugation alone has been associated with a very limited capability of cell separation and isolation. At 800 g and 1280 g, the number of nucleated cells per cc was only 10^4^ [23]. Similarly, vibrating, shaking, and centrifugation showed a relatively low percentage of progenitor cells (< 5% in 125,000 nucleated cells/cc) if compared to enzymatic methods [27].

As a matter of fact, it is indeed important to clearly understand that the simple process of adipose tissue extraction, decantation, and condensation should not be confused with all the techniques for SVF isolation and concentration. Therefore, it is inappropriate and speculative claiming, as often it is done, that simple lipofilling procedures are “stem cell therapies.”

Whenever the purpose is mainly volumetric, either for obtaining a long-lasting 3D volumetric enhancement such as in breast augmentation (Zocchi et al. [28]), gluteal augmentation (Willemsen et al. [29]), facial defects, vulvar atrophy, or to correct asymmetries or superficial irregularities originated by previous surgeries such as in breast reconstruction (Debald et al. [30]), it is not necessary to achieve the complete disruption of the adipose tissue, limiting the action to simply eliminate by centrifugation and condensation of all the unnecessary fluid components and the lipidic fraction (TGS) to ensure a better residual volume meanwhile limiting complications.

When, on the other hand, the specific aim is to induce a strong regenerative boost in the recipient site without volumetric purposes, it is necessary to further increase the level of AT disruption for extracting and concentrating all the cellular components contained in its SVF meanwhile ensuring the highest possible cellularity and vitality.

Since the beginning of the now long history of the therapeutic use of AT derivatives, more than 30 systems for SVF isolation and concentration have been developed, registered, and proposed on the market. While till recently most of them were enzymatic-based systems [16, 31] and only a few were nonenzymatic–based. Nowadays, the proportion between the two different methods has consistently, if not completely, shifted due to the fact that enzymatic-based procedures have progressively lost appeal and consensus for many reasons. The high costs of enzymatic procedures, the need to have an enzymologist in the research team for residual enzymatic level assessment in the final cells specimens and eventually, the safety concerns related to the use of enzymes outside of a laboratory facility are some of the many reasons that are pushing researchers to develop new options in favor of mechanical disruption of AT [21][21]. Moreover, collagenase has been classified as a biological drug by most of the regulatory authorities, such FDA and EMA, imposing full compliance with specific cGMP and cGLP conditions, which is often impossible for many hospitals and practitioners.

On the contrary, mechanical isolation methods of SVF are totally safe, less costly and less time-consuming and way more efficient than enzymatic procedures. In addition, some authors reported a reduced level of cell contamination after mechanical isolation when compared to enzymatic treatment [32]. The main pitfall is represented by the higher content of blood mononuclear cells, which appeared to be related to the location of ASCs precursors and pericytes in the perivascular niches, requiring releases [23, 33–36]. In the authors’ opinion, the only real advantage of enzymatic digestion using collagenase to isolate and extract the SVF from the adipose tissue is that eventually, it can offer the possibility of obtaining the tissue breakdown to the single cell. This feature can be an advantage only whenever planning to proceed with expansion and cultivation of cellular precursors in the laboratory, but these technical steps clearly and undoubtedly fall into HGM classification, not allowed by the current legal frame for therapeutic purposes in most of the countries. If it is true that at the present time, all the existing methods for mechanical isolation and concentration of the SVF from the AT do not offer this capability to isolate the single cells but only cellular clusters of different sizes, depending on the methodology, we strongly believe that this is not a limitation. In fact, on the contrary, this can be an advantage because in most of the regenerative methodologies currently in use, it is way more useful and appealing the possibility of grafting cells clusters, either macro or microclusters depending on the clinical needs, rather than single cells. As a matter of fact, maintaining their original 3D architecture and, above all, preserving the integrity of the donor site “niche” cell clusters can better support and favor the structuring and conservation of new niches in the recipient site, playing a crucial role in cells intake and proliferation [28] (Table 3).

**Table 3:** Characteristics of mechanic vs. enzymatic methods for the isolation and concentration of SVF. Collagenase and any other type of enzymes are banned from our practice since 2014

Mechanic	Enzymatic
Cells clusters only	Single-cell break-down
10–15 millions of nucleus cells × ml	5–8 millions of nucleus cells × ml
10–15% dead cells	15–30% dead cells
Preserves ECM	Digests ECM
Less AT required (less surgery time)	More A.T. required (more surgery time)
Clean and free from any bio-derivates	Animal or bacteria-derived products
Cost of the equipment $100–40,000	Cost of the equipment $20,000–90,000
Cost of disposables $30–600	Cost of disposables $300–1200
Processing time > 30 min	Processing time 90/120 min

Due to the previously described technical and legal reasons, since early 2014, the authors banned from their OR. and labs the use of collagenase for enzymatic AT digestion. In order to continue to extract all those cellular components necessary to enrich regenerative grafts, they tried countless technical options for separating and extracting SVF with very limited and unsatisfactory results. Seeking for better solutions and thanks to their long-term experiences in the use of ultrasonic energy (US) in many other surgical fields, such as ultrasonic-assisted lipoplasty [28], in early 2015, they eventually started to use the US to sonicate and emulsify the condensed AT to separate and isolate its SVF.

**Fig. 5:**
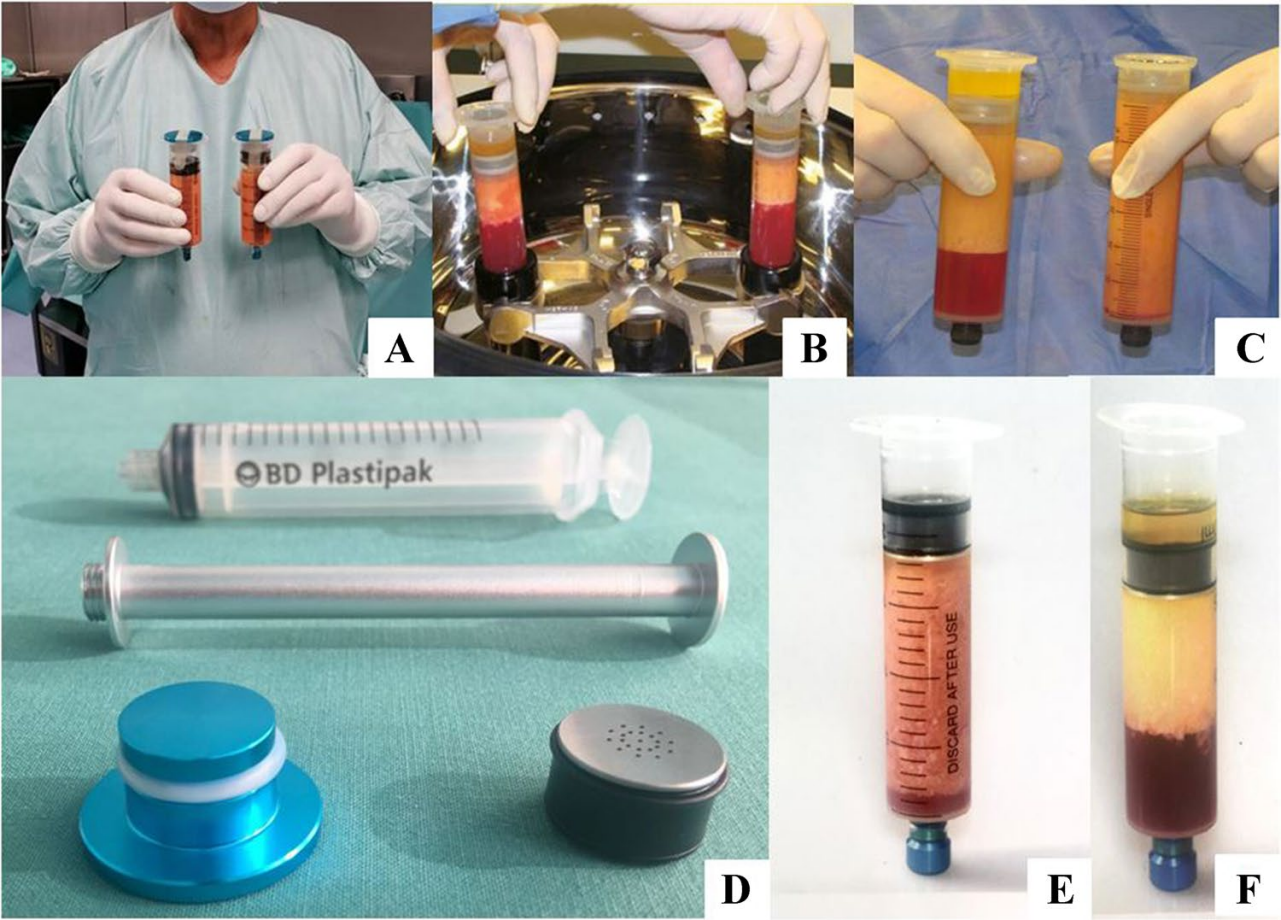
**A**, **B**, **C**, **D**, **E**, and **F** Lipocondensation the already decanted AT is undergoing to a phase of high-speed centrifugation using a dedicated system (9 min at 2300 g). Because of the centrifugal force, the heavy metal plunger of the fat processing units (FPU) apply a very high pressure (up to 130 kg × cm^2^) able to destroy the adipocytes while preserving SVF integrity and vitality. The oily fraction TGS is collected and separated in the upper part of the FPU and can be easily discarded. **A**, **B**, **C** Lipokit using special fat processing units (FPU) (Medikan–Korea).** A** Decanted fat; **B** condensation process; **C** comparison of fat before and after condensation process with lipokit; **D** newer and cheaper custom made devices using simple Luer-Lok syringes; **E** before the condensation process; **F** after the condensation process in normal syringes

In this procedure, known under the name cellication, the already decanted fat needs to undergo a process of lipocondensation. The AT is transferred in specially conceived high-resistance syringes called fat processing units (FPU) to undergo a high-speed centrifugation phase (2300 g for 9 min) using a special device (Lipokit-Medi-Khan, Seoul, Korea) in order to separate the fluid fraction (water, blood, and TGS) from the dense cellular and ECM fraction and to compact the volume (Fig. 5).

During this first step, many but not all adipocytes are destroyed. In order to achieve the complete disruption of all remnant adipocytes while preserving the SVF and the integrity of the staminal component, the condensed fat is emulsified with ultrasonic energy for 30 s using a new generation ultrasonic generator with a dedicated titanium probe (LipoSaver-LHbiomed, Korea).

**Fig. 6:**
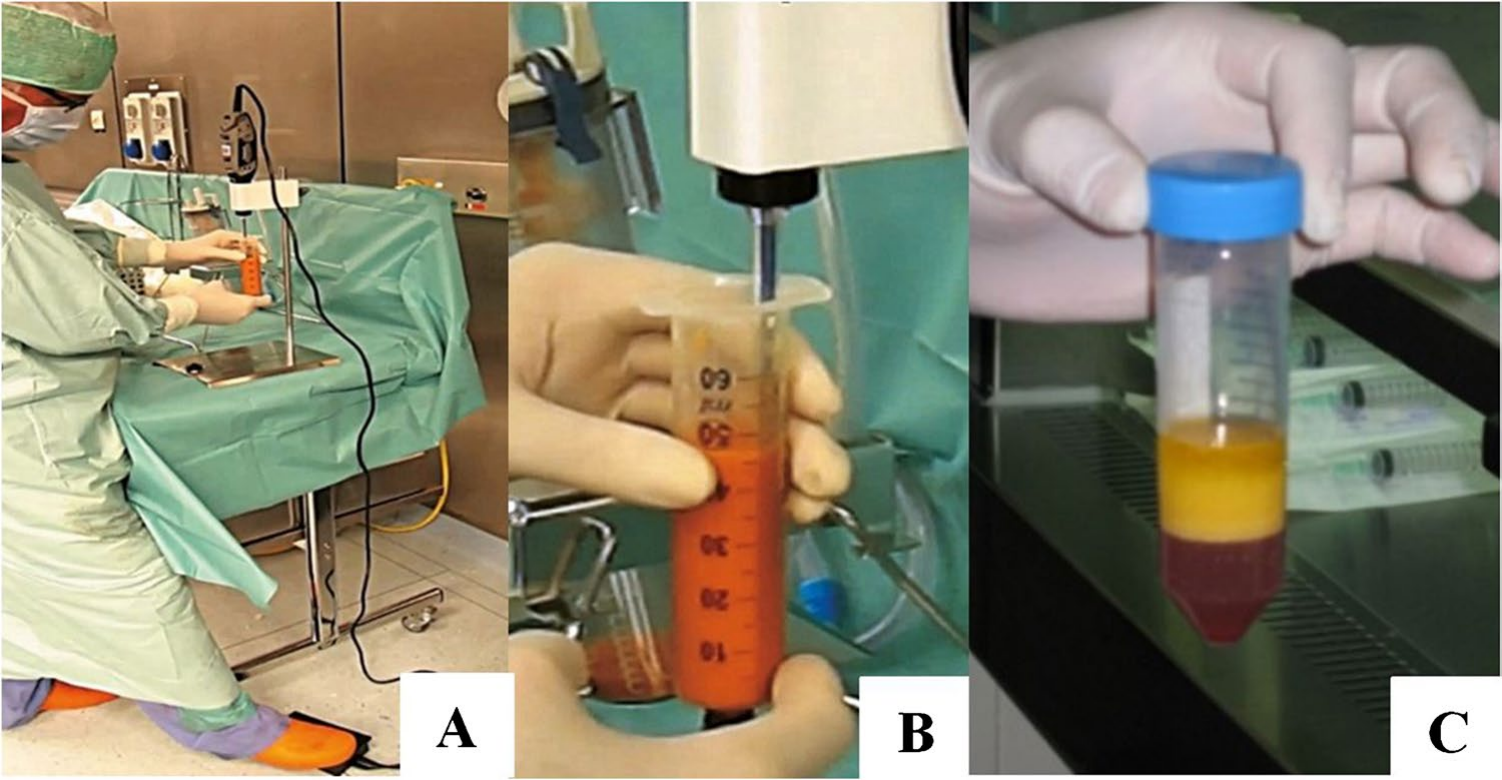
**A**, **B**, and **C** Cellication; **A**, **B** the condensed fat is sonicated with a special titanium probe for 30 s. This action disrupts any remnant adipocytes; **C** after a final low-speed centrifugation (600 g for 2 min) the cellular components are collected in the lower part of the vial

The sample is then submitted to a quick phase of low-speed centrifugation (600 g for 2 min) for obtaining the separation of the different components (Fig. 6). The cellular SVF (CSVF) is still intact after these subsequential manipulations and deposits in the lower part of the processing tube and it can easily be removed either by micropipetting or by direct transfer [37, 38].

The authors have used this technique as an elective method for SVF isolation and extraction for more than 3 years. However, few concerns should be highlighted: the final cellular fraction that can be obtained represents less than 2% of the total volume of the original condensed fat and its cell sorting has been assessed to be 15–20% poorer than the one obtained after enzymatic digestion, with average cellularity close to 300,000 MSC precursors per cc of SVF and an average viability close to 89% [28]. The still limited efficiency for SVF isolation, the very high cost of the related surgical equipment (lipocondensation centrifuge and ultrasonic generator), the long duration of the processing and the potential risks of contamination of the grafts during the manipulation have always represented the most important limits of this technology.

Still unsatisfied with the results and seeking for more efficient and more affordable alternatives, the authors never quit to carefully try all the newest mechanical procedures, both manual and automatic, for the separation, isolation, and concentration of SVF [16]. Even if, on one hand, automatic and semiautomatic methods are reducing graft air exposure and limit risks of processing deviation related to the human factor, they are, on the other hand very (sometimes extremely) expensive and they do not allow any customized use from preset programs, de facto clipping the wings of surgeons’ personal fantasy and style. Therefore the authors mainly focused their attention and efforts on nonautomated systems.

The main parameters used to assess their efficacy were efficiency for isolating nucleated cells and concentrating progenitor cells, versatility, speed, and cost. In recent years, authors have tried more than 15 mechanical devices for the extraction and concentration of the SVF. Some of those turned out to be totally useless and inefficient, some others instead allowed to obtain an acceptable macro-fragmentation of the AT. However, the cellularity and vitality of the final product were still far from the ones they managed to obtain with enzymatic digestion and the related cost was still excessive. This stimulated the authors to continue their research path with obstinacy in order to improve techniques and efficiency.

### Microlyzation

In early 2018, as part of this long path of research and clinical trials, the authors have been requested to evaluate the first prototypes of a new blunt force–based method for macro- and micro-fragmentation of AT (Microlyzer™ T-Lab,Turkey). Since the very first approach, this new system looked to be a valid option, especially for treating small to medium amounts (80–100 cc) of tissue, which is the most common need in the majority of clinical situations. It is definitely a cost-effective, user-friendly and fast micro-fragmentation system based on sharpened edge microblades of different sizes (2400, 1200, and 600 μm) with a special and exclusive honeycomb design. After trying them and carefully testing both, nucleated cell number and vitality and ADSC progenitor characterization, authors realized that probably the long-time sought solution was approaching.

**Fig. 7:**
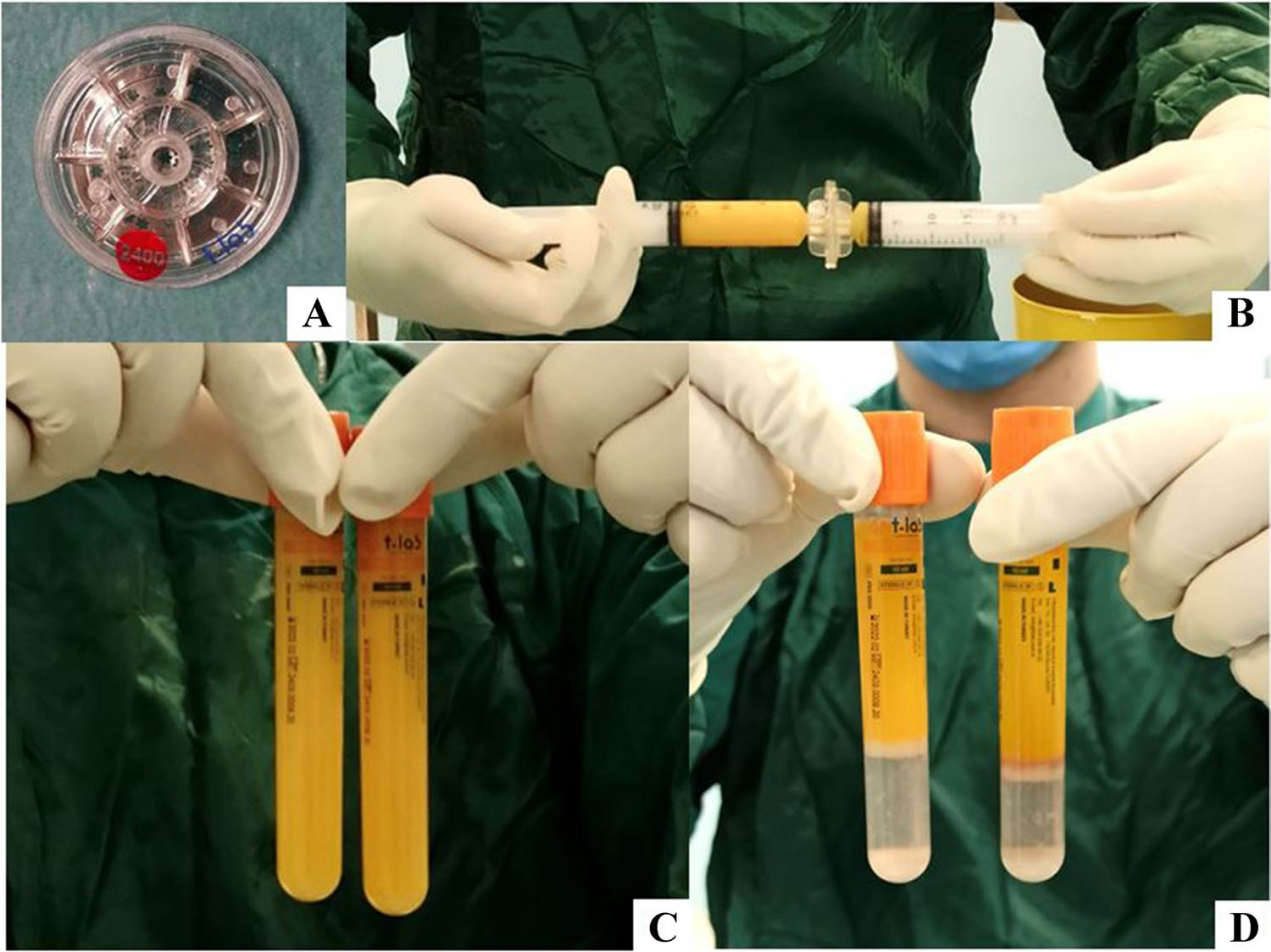
**A** The cartridge of Microlyzer has two female-to-female Luer-Lok adapters of different sizes (2400 µ, 1200 µ, and 600 µ). **B** The tissue has to be transferred between the two syringes from 3 to 5 times across the blades. The blades must be used from the largest one to the smallest one until the desired consistency and viscosity are achieved; **C** AT before the treatment with MycroLyzers; **D** cell component separation after low-speed centrifugation 650 g for 3 min. Cellular SVF deposits in the lower part of the processing tube and it can easily be removed either by micropipetting or by direct transfer

After lipoaspiration, the centrifuged AT sample is shifted 3/4 times between two 10 cc Luer-Lok syringes through the Microlyzer from the largest to the smallest size. In this way, a fresh micronized fat with high consistency and viscosity properties is obtained (Fig. 7).

To complete the process of microlyzation the AT sample should undergo a sequential tissue disruption with all three microblade sizes, 2400, 1200, and 600 μm, and a final phase of low-level centrifugation (450 g for 3 min). In order to get the horizontal stratification of the layers for a homogeneous separation of the different components, it is mandatory to use a swinging-bucket centrifuge.

The microlyzation process has shown a high capability to selectively disrupt the adipocytes without damaging key regenerative components and allowed isolating a SVF with a range of 15 to 18 million nucleated cells per cc of SVF with a range of 5 to 7% of precursors [16].

From April 2018 to June 2019, more than 200 tests on AT samples harvested from different body areas were performed. Three years later, after a complex process of improvements and changes, we can say that now we can rely on a truly efficient micro-fragmentation system and we are very satisfied with the constant qualitative result of the final cellular product.

**Fig. 8:**
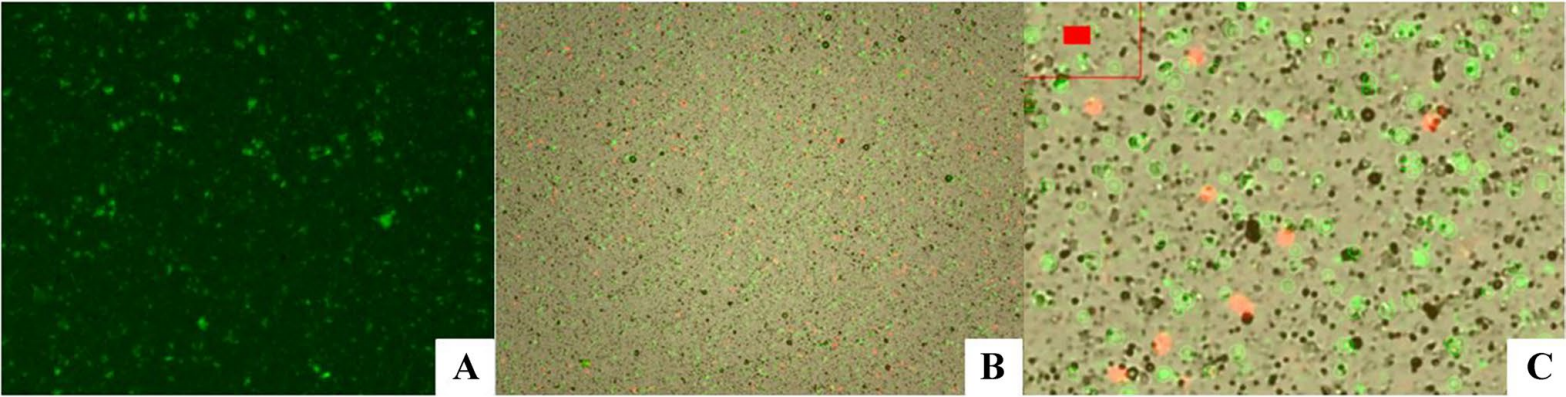
Microlyzation extracts the cellular component of the adipose tissue without damaging key regenerative components as reported by the nucleated cell counting; **A** live cells (green fluorescent); **B** live cells (green fluorescent), dead cells (red fluorescent), and other fractions (e.g., ECM and cells residuals); **C** zoomed part of image B. Detailed view of live and dead cells and other fractions

**Fig. 9:**
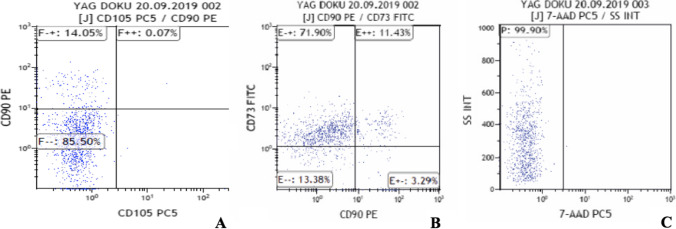
Characterization is carried on by considering the total number of fresh cells, no cultured or expanded to define the different types of cell population and recognize markers of mesenchymal stem cells, **A** characterization with CD105; **B** characterization with CD90; **C** this method allows to obtain up to 600,000–800,000 MSCs/ml (mean 700,000) with a cell viability of 99.9%

Microscopical analysis showed that microlyzation allows to obtain a very homogeneous AT macro- or micro-fragmentation (depending on the clinical needs) with a high concentration of ADSCs precursors (650,000–800,000 MSCs/ml) with cell viability of 99.8%, recognizing CD90 and CD105 positive cell markers for mesenchymal stem cells. In addition, the cell adherence after the “3-day” cycle was as high as 60% in the isolated cell population [16] (Figs. 8 and 9).

These results are almost comparable to those obtained from enzymatic manipulations and they are in strong support of microlyzation technique with the aim of creating regenerative mixtures with minimal mechanical manipulation with the highest cell concentration from fresh AT samples.

Similar mechanical separation devices (Adinizer™-BSLrest, Busan, South Korea) are now also hitting the market. However, in the authors’ hands, the procedure required a longer manipulation due to the lower number of blades and its lack of honeycomb technology and cell sorting show lesser efficiency.

For this reason, Microlyzers are today a technique of choice for isolation of SVF from adipose tissue to be used for all those different clinical applications in different specialties where a strong and reliable regenerative action is required. However, the final cellular products obtained are not standardized and can greatly differ for cellularity, viability, and characterization.

As a matter of fact, the phenotypic characterization of adipose-derived tissue cell precursors can show significant differences from one patient to another and from one anatomical area to another. Literature suggests that, except for donor areas, gender, race, age, body structure, and fatty component pathophysiology profile (hypo/normo/hypertrophic) can be strong determinants of the quality of the ASCs. It’s a common finding, for example, that younger patients own a pool of ASC precursors with a greater proliferative capacity (and higher phenotypic expression for surface markers) than those from older patients [39]. In the authors’ experience, flow cytometer data revealed high variability among patients and anatomic areas of CD105 ( +) CD45 ( −) cells even using the same technique for extracting SVF.

Following these basic concepts and in the aim to obtain more consistent results, it is necessary to establish a minimum threshold value of nucleated cells and MSC precursors (such as 4–5 million nucleated cells per cc of SVF with 4–7% of precursors) to consider a regenerative procedure effective. For this reason, each specific method of SVF isolation and concentration should report the minimum and maximum range (worst and best scenario) of its real efficiency in isolating and concentrating nucleated cells (i.e., from 5 to 15 million nucleated cells per each cc of SVF).

Hopefully, in the very near future, there will be the obligation of establishing a real ID card (as already required for other surgical procedures i.e., breast implants) for each regenerative therapy, reporting donor and recipient areas, type of cellular components, and precursor cellularity and vitality (Table 4).

**Table 4:** Template for the ID card of REMIX regenerative procedures. Realizing cells precursors phenotypic characterization for each procedure can be expensive and complicated. Manufacturers should provide clear and reliable data on mean performances of their SVF isolation devices

Patient ID cards for REMIX regenerative procedure				
Name				
Gender	F		M	
DOB				
REMIX type				
Donor area				
Recipient area				
AT type	Hypotrofic	Hypertophic	Hypoplastic	Hyperplastic
Nucleated cells				
Cellularity				
Viability				
ADSC precursors				

### Blood components

The second key component of the BACTs is represented by blood components (BC) containing the patient’s own blood platelets, which act as a natural source of specific and aspecific GFs (e.g., PDGF, IGF, VEFG, PDAF, TGF-beta, and many others). The release of GFs is triggered by the activation of platelets and can be kick-started by different substances such as thrombin, calcium chloride, and collagen. Blood-derived GFs support chemotaxis, proliferation, differentiation, and angiogenesis in a highly controlled way by releasing different molecules (e.g., fibronectin, vitronectin, and sphingosine 1-phosphate). However, different compositions of BC type offer different features, GF concentrations, and therapeutic actions [40, 41].

Red and yellow platelet-rich plasma (PRP), high-concentration amber PRP (HCAPRP), plasma rich in growth factors (PRGF) platelet lysate (PL), or platelet-rich fibrin (PRF) are the most commonly used blood components [42, 43].

Among them, red PRP has been widely used in the past, given its easy extraction method and affordable cost. However, its richness in red and white cells is at the origin of significant cytokine activation and enhancement of local inflammation at the point that it can be even contraindicated in some specific pathologies, such as osteoarthritis (OA) type 1 where the increased inflammatory response can worsening the clinical situation.

PRGF and PL can be considered as enhanced types of BC, with a higher pool of GFs, if compared to red or plain amber PRP, containing a higher quantity of GFs and inducing a better anti-inflammatory response. PRGF and PL can be added to freshly isolated SVF whenever a high concentration of regenerative elements inside of a small volume is required. However, even if the clinical use of PRGF and PL is certainly more valuable and efficient than red or plain amber PRP, these BC are still less commonly used due to their very high and often unjustified cost of the isolation device and of the related kits (Magellan/Regennex®).

**Fig. 10:**
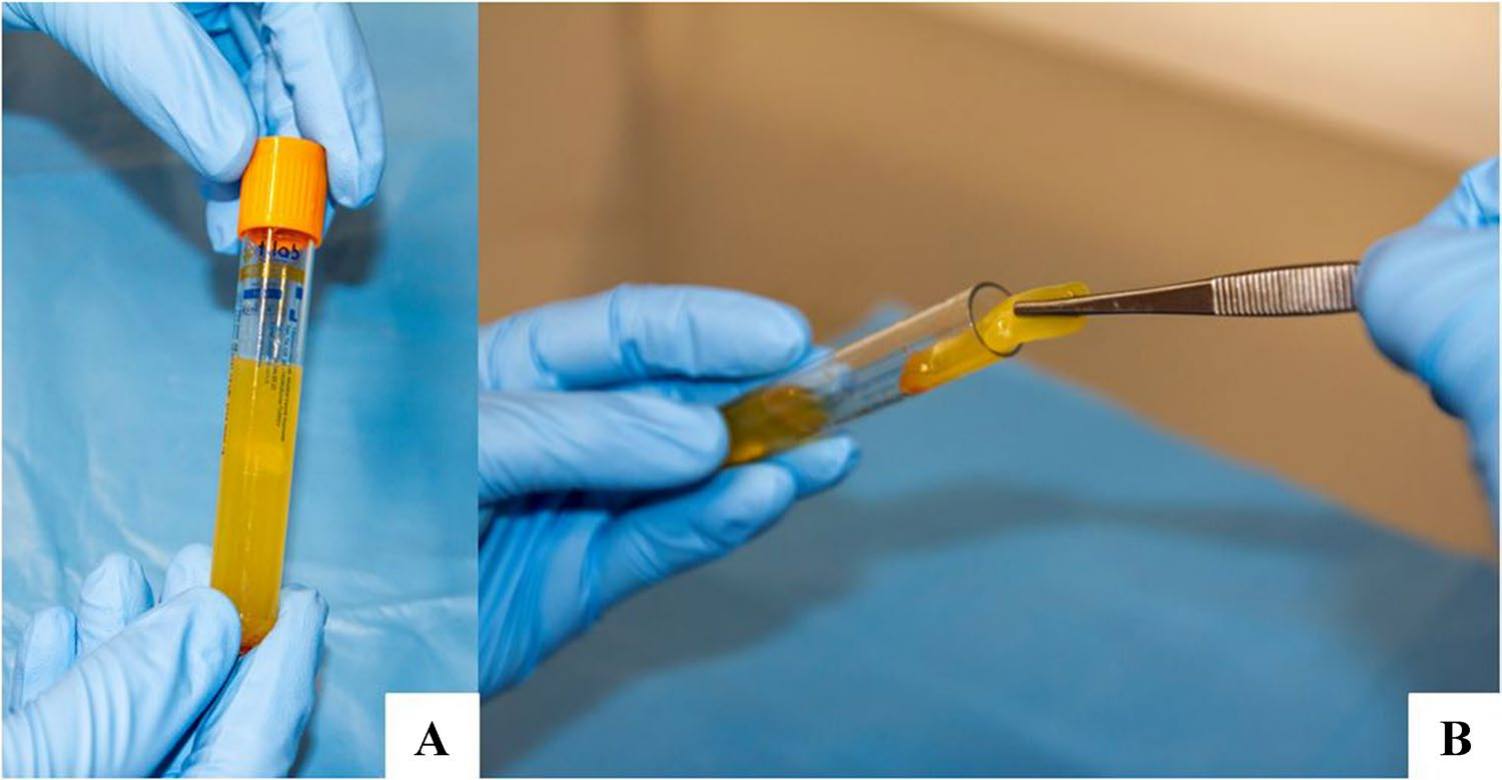
**A** Condensed PRF,** B** 3D mesh of PRF isolation can be obtained just by waiting 40 min after a third spinning step of 8 min at 850 g

It is the authors’ modest opinion that the most appealing and innovative BC available today is certainly represented by PRF, a slow-release autogenous matrix fibrin gel product that can be considered a three-dimensional (3D) structure that favors the delivery and support of cell sheets. Unlike platelet-rich plasma, PRF can be obtained from the patient’s blood simply by using repeated cycles of low-speed centrifugation without adding any type of chemical or anticoagulants. The procedure used by the authors does not require any special automated device and expensive kits but just a simple system using a centrifuge a very inexpensive kits (Next PRP and PRX by T-Lab-Turkey). In less than 15 min and with minimal cost, from 20 cc of blood it is possible to obtain from 4 to 6 cc of jellified PRF only alternating repetitive spins (from 3 to 5) at different G and duration without using any chemical agents. Whenever necessary, it is also possible to obtain a 3D PRF mesh just by adding further spinning steps alternated to decantation phases (Fig. 10).

Cytokines, GFs, and cells are mixed together in a homogeneous multifactorial pool where the different factors can be slowly released from PRF over time (from 3 to 18 days). PRF is very rich in EGF, FGF, and specific pro-inflammatory cytokines such as IL-1b, IL-6, and TNF-alfa and through a strong secretomic action, it is able to affect the genetic and cellular activity, playing a significant role in the inflammatory response of the grafted biomaterial to the recipient site [44].

### Bio-catalyzers

A complete cell culture medium is composed of a basal medium containing low-molecular-weight substances such as inorganic ions, amino acids, vitamins, and other additional components (e.g., glucose, pyruvate). Due to the fact that medium composition is often inadequate for the in vitro homeostasis of different cell lines, high-molecular-weight supplements (e.g., proteins) have to be added to fulfill cell requirements. However, even if confident of the intrinsic safety of this strategy, because of the still unclear oncogenic role of cancer stem cells, in some oncological patients we are limiting the use of high-molecular components.

There are two different types of bio-catalyzers, which can be added to the BACTs (Table 5).

- Type 1: amino acids (AA) and vitamins (VITs) reproduce the action of a DMEM while reduced glutathione (GSH) antagonizes ROS deleterious activity.

**Table 5:** Amino acids, vitamins, GSH, and high-molecular-weight morphogenetic proteins are added to the regenerative mixture to support the cell’s take and survival in the recipient site

Biocatalyzers	Type	Action
Type 1	Amino acids, proteins, GSH	Ability of mimicking in vivo the action of DMEM medium
Type 2	Morphogenetic proteins	Stimulation and redirection of cells toward specific cellular lines

#### Amino acids

AA improves and support protein synthesis, presenting a key role in mammalian cell cultivation and homeostasis [45]. With the aim of mimicking the supportive role of a DMEM it is useful to enrich BACTs with a pool of 12 essential L-amino acids such as arginine, cysteine, leucine, isoleucine, lysine, methionine, phenylalanine, threonine, tryptophan, histidine, tyrosine, and valine. These additional components working in synergism with the other components support cells’ intake and growth to the host recipient site [46].

#### Vitamins

VITs act as cofactors or prosthetic groups of different enzymes, with essential roles in both cellular and molecular functions. The most active vitamins are biotin, folate, nicotinamide, pantothenic acid, riboflavin, thiamine, and vitamin B12. Although the necessity of very low concentrations of this component, the presence of VITs in vitro is key and their absence may lead to decrease cell growth, death, or loss of function. Therefore, we add vitamins to our BACTs in order to improve local cell homeostasis and survival [47].

#### Reduced glutathione (GSH)

GSH is one of the most powerful antioxidants in nature and can support cell survival antagonizing the negative effect of reactive oxygen species (ROS) on grafted ADSC precursors. Other means to oppose the oxidative stress on cell grafts, such as deferoxamine and N-acetyl cysteine, has been also proposed [48, 49], but GSH is the most important and versatile endogenous ROS scavenger [50]. Used in the past in specific clinical situations, such as acute poisoning and severe postoperatory stress, both per os and IV administration. Today it is often used off label as ancillary therapy in the most different cases from cataract and glaucoma to skin whitening. The authors proposed a novel use by directly adding GSH to the BACTs mixture (200 mg in 2-ml diluent) in order to counterbalance the oxidative stress occurring in the recipient site after the grafting.

- Type 2: morphogenic proteins.

Bioactive morpho-proteins can be added to BACTs to stimulate cells’ recruitment and redirecting toward specific cellular lines and to support their intake to the host recipient site. For the clinical treatment, for example, of osteoarthritis type 2, the addition of 1500 IU of bone morpho protein 2 (BMP-2) to BACTs drives ADSC precursors toward the chondrogenic lineage, boosting the cartilage regeneration for the clinical treatment [51]. This possibility could be very interesting, especially for those cases in which the regenerative action should be focused toward a specific tissutal target.

### Carriers

Carriers have the ability to improve cellular structure and graft’s implant. The HA (hyaluronic acid), the PA (polycaproic acid), and the ECM (extracellular matrix) represent important carriers within this field. The HA has a fundamental role in tissue volumization and regeneration. At the beginning of our in vitro works, we mainly used a buffer-type HA (pH > 7.1) because the traditional HA with a pH between 6.6 and 6.7 was negatively interfering with niches’ preservation and function. Even the PA CH_3_(CH_2_)_4_COOH, an hexanoic acid derived from the hexane (pH = 7), was proven to be effective. However, we have never obtained satisfactory results with HA or with PA.

At present, the recipient ECM is the main carrier of our BACTs. Its three-dimensional network is composed by collagen, enzymes, and glycoproteins. Due to its features in cell adhesion, cell-to-cell communication and differentiation, the ECM currently represents the most reliable and user-friendly tool to facilitate the reimplantation of the graft while supporting the structure of the other bio-active components.

### The REgenerative MIXture (REMIX)

It is authors’ opinion, now more and more accepted, that at the present day and with the current knowledge in this field, it is unlikely to support the old concept “one-graft-fit-all” and that type and quality of the regenerative grafts should be substantially different and adapted to anatomic areas and clinical indications. Therefore, also all the related strategies and methodologies for their preparation should be different, but so far, precise standards and protocols for customized preparation of advanced regenerative grafts has not been established.

### Clinical application

Translating the in vitro technical steps into the clinical daily setting for the scope of regenerative action authors, in close collaboration with many other specialists, have structured different novel protocols, using different biocomponents concentration and proportions, to be applied in many clinical fields and specialties. The different protocols are named REMIX (acronym for regenerative mixture) and are identified by specialty and type of the composition (i.e., REMIX PS type 1/2/3 or REMIX OS type 1/2); the cellular components (SVF and PRF) are expressed in percentages while the other additional components (AA, vitamins, GSH) are expressed in mg × cc. Plastic and reconstructive surgery (PS) has been the very first field of application, for breast reconstruction, microsurgery, wound healing, diabetic foot, and burns sequela. Orthopedics (OS), rheumatology (RM), uro-gynecology (UG), dermatology (DM), and pain management (PM) are some of the many other surgical specialties getting great benefits from concrete clinical applications of BACT strategies. Plastic surgeons’ skill and knowledge regarding fat harvesting and grafting is playing a crucial role for ensuring the most efficacious and customized regenerative mixture preparation in the total respect of donor areas’ integrity and safety standards. However, in the aim of obtaining the best possible results, ensuring the highest safety to the patients, this type of procedures should be carried out by a multidisciplinary team where clinical indications and grafting procedures into the different recipient sites are performed by each appropriate and trained specialist. The final section of this article is dedicated to a quick review of some clinical applications, from the most well consolidated, such as wound healing, to the newest ones, such as for the treatment of post COVID-19 interstitial pulmonary fibrosis, using different types of REMIX protocols.

### Wound healing

The stromal vascular fraction (SVF) and blood components (PRF) are usually mixed in a ratio of 5:1 (e.g., for 60 cc of regenerative mixture, 50 cc of SVF, and 10 cc of BCs). Changes in this proportion are possible according to the clinical needs. The two main aspects that influence the choice of cell proportions are the volume of the recipient site and the inflammatory state. The smaller the volume of recipient site, the higher the need for cellular component concentration. On the contrary, the more severe is the inflammatory condition, the higher is the need of increasing the concentration of GFs. In case of large volume defects, BACTs should be associated with condensed AT in order to associate a volume replacement effect to the regenerative action [52]. The REMIX type 2 for wound healing is composed of 70% of freshly insulated SVF, 20% of PRF, 25 mg × cc of amino acids and 150 mg x cc. of vitamins (Table 6).

**Table 6:** After the isolation of the stromal vascular fraction and the preparation of the other components, the BACTs are ready to be injected into the recipient site

REMIX PS type 2 for wound healing	
SVF	70%
PRF	20%
Aminoacids	25 mg × cc
Vitamins	150 mg × cc

**Fig. 11:**
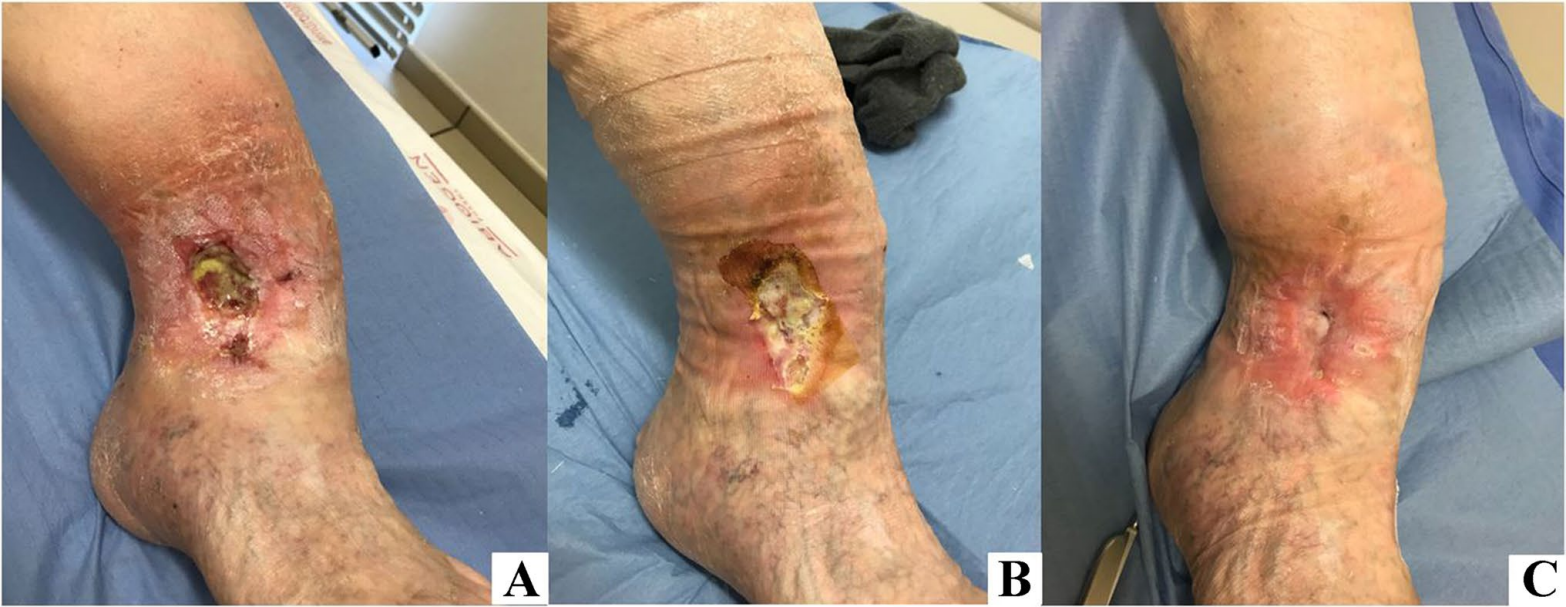
BACTs REMIX PS type 2 for wound healing (20 cc.) injected in a patient suffering from a diabetic foot ulcer supported by a nonocclusive biodress; **A** preoperative picture; **B** 15 days post-op; **C** 90 days postop (courtesy of A. Balbiano)

These components are mixed together and reinjected (in a centripetal way 0.5 cm from the lesion rims) to the host recipient site. The support of a nonocclusive biodress is highly recommended. After 6 days, we can obtain a very positive healing activity with increased local granulation. Even diabetic-foot patients could benefit from BACTs (Fig. 11).

### Regenerative-assisted post-bariatric surgery (RAPBS)

Post-bariatric surgery benefits of regenerative protocol both through volumetric and regenerative procedures. In fact, autologous fat grafting allows improvement of body contouring procedure through adipose tissue redistribution [28, 29]. Furthermore, dedicated BACTs obtained from AT disruption and SVF extraction permits to highly stimulate vascularity and regeneration, limiting the risk of peripheral necrosis and healing difficulties typical of post-bariatric patients [53, 54].

An undoubted advantage is that significant quantities of adipose panniculus are removed with lipectomy and liposuction in all post-bariatric surgery procedures. As previously described, dermolipectomy specimens can be used as sources of regenerative cellular components and processed in order to obtain autologous injectable grafts [55]. Instead of eliminating the removed fatty deposits, they can be fragmented by means of a special grinder and the material collected in 60-cc Luer-Lok syringes closed at both ends and then centrifuged for 3 min at 850 g. This phase of manipulation of the AT and the concentration of the cellular components necessary for the preparation of the regenerative cocktail is carried out while the surgery continues without lengthening operating time.

The improvement of vascular health and flap vascularization widely described in the literature is a strong support to consider the application of RAPBS in the clinical practice [56].

### Regenerative-assisted microsurgery (RAM) and regenerative-assisted supermicrosurgery (RASM)

In the last decades, microsurgery has become a reliable and effective discipline fundament to address many complex reconstructive problems. Still, surgeons are dealing with issues related to local and systemic reaction to the surgery and flap survival.

The synergic action of regenerative strategies will further extend the horizons of this exciting specialty (Zocchi 2021 [18]). In fact, they could allow a better control and limitation of the host inflammatory and immune response through cytokine modulation; they can support cell intake and differentiation and stimulate a strong action of neo-vasculogenesis, angiogenesis, and lymphangiogenesis. In addition, the strong modulation of cytokines activity, in particular of IL-6 and IL-6A obtained through regenerative components will allow to control and limit the inflammatory response resulting from the surgical trauma and the related physiochemical metabolic stress of the surrounding tissue both in autologous and heterologous tissue transplantation [57, 58].

BACTs can be applied in the field of microsurgery in three different phases:

1. Four to 6 weeks before elective surgeries to prepare and precondition the donor areas of free flaps to increase the harvested area improving the vascularization through choke vessels.
2. Intraoperatively to reduce and modulate the immediate inflammatory response linked to the activation of the cytokine pools trough slow releasing of specific GF meanwhile ensuring a “healing activity” sustained by pericytes.
3. In the postoperative period to modulate inflammation and improve angiogenesis and lymphangiogenesis in the recipient site, accelerating the flap autonomization, reducing peripheral stasis and ischemia supporting the cellular recruitment toward specific cellular lines and cells intake.

The preliminary evaluation of the results obtained following the grafting of dedicated BACTs during the final phase of the microsurgical procedure, mostly at the level of the boundaries between flaps and the recipient areas, clearly stands for a better control of the inflammatory response and an increased neoangiogenesis activity supporting the intake and the integration of the flap itself [56].

Further studies will be performed to elucidate the therapeutic value of using regenerative strategies to consolidate outcomes and results.

### Orthopedic surgery

Orthopedic surgery is definitely the surgical specialty that can benefit most of these new therapeutic strategies. At the present time, the use of basic and advanced regenerative procedures in orthopedic surgery is estimated close to 30% of the total number of procedures globally performed (3 times more than in plastic surgery). The possibility to improve joint mobility and pain control stimulating cartilage regeneration in OA types 1 and 2, or the possibility to speed up recovery and healing processes after acute or chronic trauma and tendons reconstruction or to improve bone consolidation in complex fractures is concrete. The REMIX ortho type 1 is composed of 80% of freshly isolated SVF, 20% PRF, 1500 IU of BMP2 every 10 cc of sample, 25 mg × cc of amino acids and 150 mg × cc. of vitamins and improves joints regeneration. Whenever it is necessary to stimulate a chondrogenic cell redirection, it is possible to add 1500 IU of bone morpho protein 2 (BMP2) to the regenerative mixture. These components are mixed together and grafted under echo guidance to the recipient site (Table 7).

**Table 7:** The components are mixed together and grafted under echo guide into the recipient site

REMIX ortho type 1 for joints regeneration	
CSVF	80%
PRF	20%
BMP2	1500 IU
Amino acids	25 mg × cc
Vitamins	150 mg × cc
GSH	g × cc

**Fig. 12:**
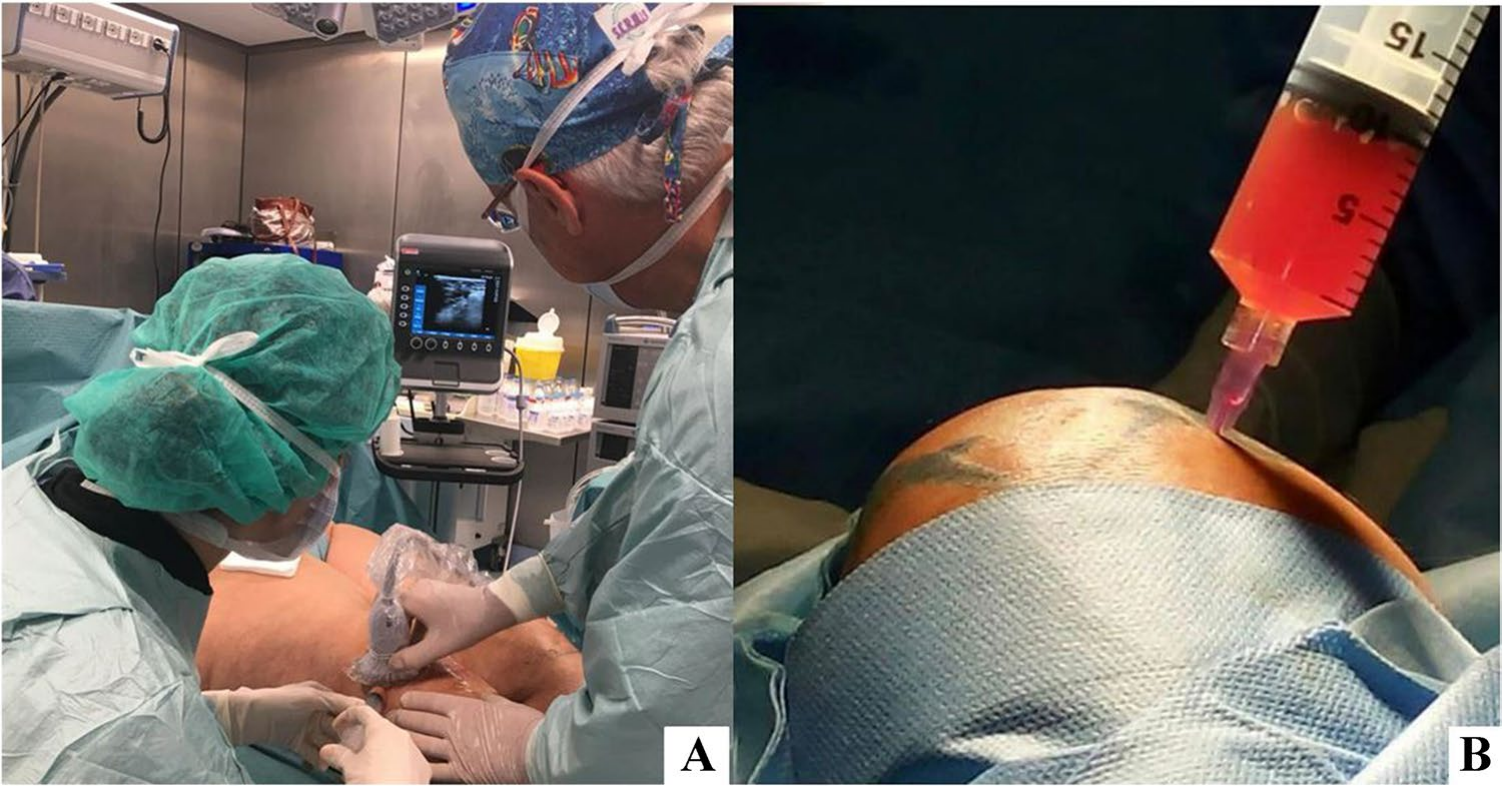
Knee joint infiltration **A** ultrasound guided injection; **B** detail of intra-articular injection (courtesy of T. Nguyen)

We recently performed a comparative study on patients affected by bilateral knee joint osteoarthritis. A total of 22 patients were treated on one side with centrifuged SVF, whereas the contralateral side was treated with BACTs: the REMIX ortho type 1 (Fig. 12).

We assessed pain control and joint function at 30, 90, and 180 days after local treatment by using the VAS pain score and WOMAC osteoarthritis index. A significant improvement in both pain control and joint function in the side treated with BACTs has been clearly demonstrated in our first clinical attempts (Table 8).

**Table 8:** Comparative study on 22 patients suffering from knee joint AO type 1/2: patients were treated on one side with fresh adipose-derived SVF and on the contralateral side with the REMIX ortho type 1: pain control improvement assessed by VAS Scott–Hutchinson scale; function improvement assessed by WOMAC Osteoarthritis index

	SVF vs. REMIX knee osteoarthritis type 1	
	SVF	REMIX
30 days	31% pain control improvement	53% pain control improvement
	23% function improvement	39% function improvement
90 days	36% pain control improvement	62% pain control improvement
	29% function improvement	44% function improvement
180 days	36% pain control improvement	73% pain control improvement
	30% function improvement	53% function improvement

### Rheumatology

Rheumatology is a very promising field of BACT application. The most common indications are for treatment of hands and peribuccal sclerodermia, rheumatoid arthritis, Reynaud’s disease, and lupus sequela.

BACTs REMIX rheuma type 1 for hands and peribuccal sclerodermia are composed of 50% of condensed enriched AT, 30% of SVF, 20% of PRF, 15 mg of amino acids for every cc of the total graft volume and 50/75 mg × cc of vitamins (Table 9).

**Table 9:** The additional action of amino acids and vitamins allows to obtain a strong regenerative action and improve scleroderma signs and symptoms

REMIX rheuma type 1 for hand sclerodermia	
Condensed enriched AT	40%
SVF	30%
PRF	20%
Amino acids	15 mg × cc
Vitamins	50/75 mg × cc

**Fig. 13:**
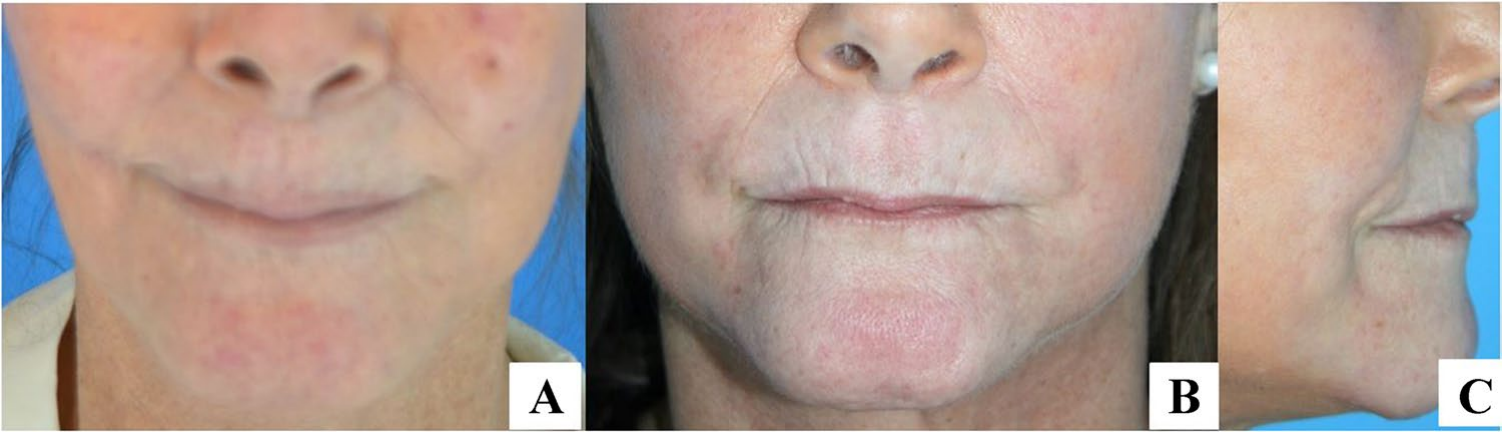
The Rheuma type 1-BACT components and the concomitant CO_2_ pneumodissection of the peribuccal area induce a local regenerative microenvironment. **A** preoperative image; **B,C** postoperative images at 6 months (courtesy of G. Blugerman)

**Fig. 14:**
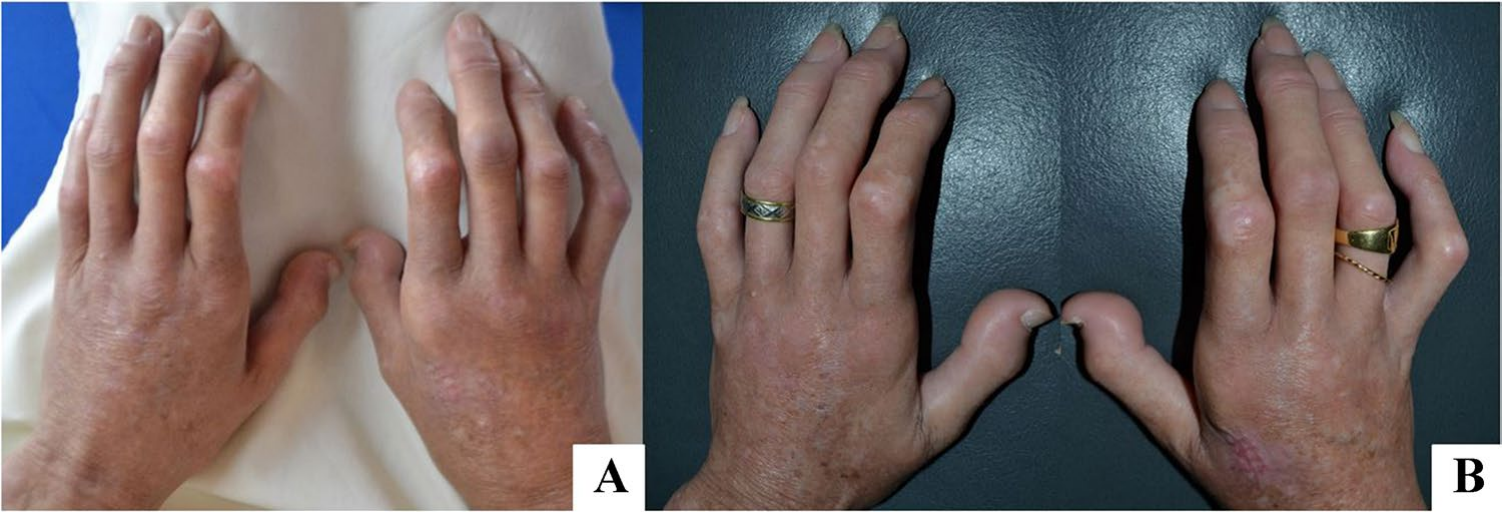
The Rheuma type 1-BACT components and the concomitant CO_2_ pneumodissection of the hands and fingers induce a local regenerative microenvironment. **A** Preoperative image; **B** postoperative images at 6 months (courtesy of G. Blugerman)

After the preparation of the AT and of BCs, the components are mixed together and grafted to the recipient site, in this case on both hands. Given the impairment of the vascularization of the recipient site in this group of patients, recipient bed preparation and preconditioning with CO_2_ pneumo-dissection play a crucial role in supporting local regeneration. Blugerman described this technique for the first time in 2016 [59]. CO_2_ pneumodissection is essentially based on the pressurized expansion of the recipient site with carbon dioxide. The simultaneous vacuum application facilitates the graft implantation, homeostasis, and integration through the enhanced CO_2_ levels and Bohr effect. Finally, the bioactive REMIX has to be grafted in situ (Figs. 13 and 14).

### Gynecology and urogynecology

Aesthetic gynecological imperfections have been always a difficult problem to deal with, above all because they concern the psychological female sphere. We are currently testing regenerative vulvar rejuvenation and vaginal regeneration procedures. BACTs represent an important tool even to treat incontinence and clitoral insensitivity.

The REMIX gyno type 1 is composed of 70% of condensed enriched AT, 20% of SVF, 10% of PRF, 25 mgs × cc of amino acids and 100 mg × cc of vitamins and 20 mg × cc of GSH (Table 10A) and it is mainly used for vulvar reshaping and augmentation.

**Table 10:** (A, B) The condensed enriched AT, SVF, and PRF is carefully added to the BACTs. The addition of amino acids and vitamins improves regenerative action

A. REMIX gyno type 1 for vulvar reshaping	
Condensed enriched AT	70%
SVF	20%
PRF	10%
Amino acids	25 mg × cc
Vitamins	100 mg × cc
GSH	20 mg × cc
B. REMIX gyno type 2 for vulva and vagina regeneration	
SVF	50%
PRF	50%
Amino acids	50 mg × cc
Vitamins	150 mg × cc
GSH	40 mg × cc

**Fig. 15:**
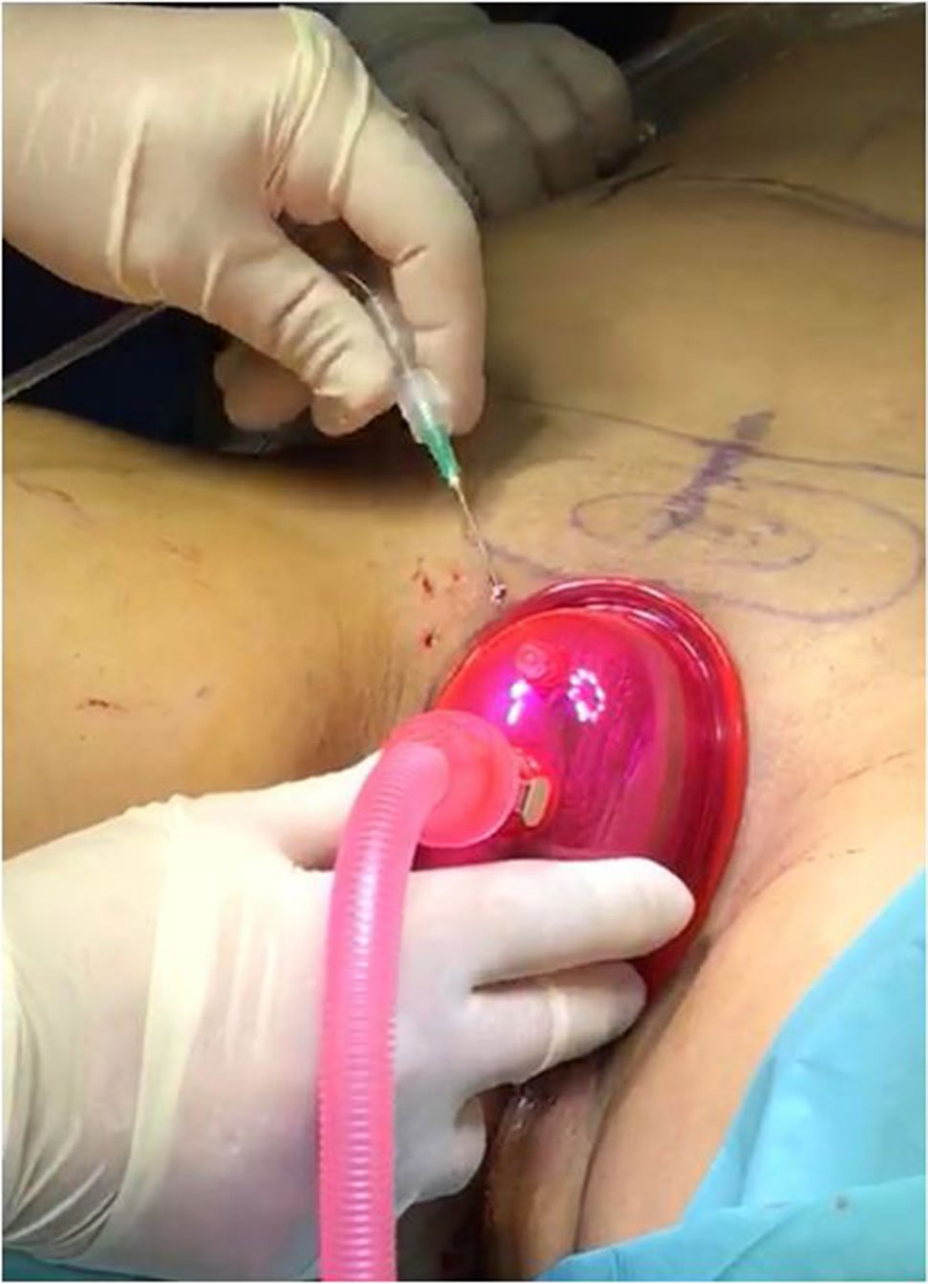
The CO_2_ pneumodissection and simultaneous vacuum application favors the preparation of the recipient site

As pioneered by Blugerman, the CO_2_ pneumodissection and simultaneous vacuum application of the recipient site before injection facilitate the metabolic reintegration of cell components trough induced hypercapnia and Bohr effect (Fig. 15).

**Fig. 16:**
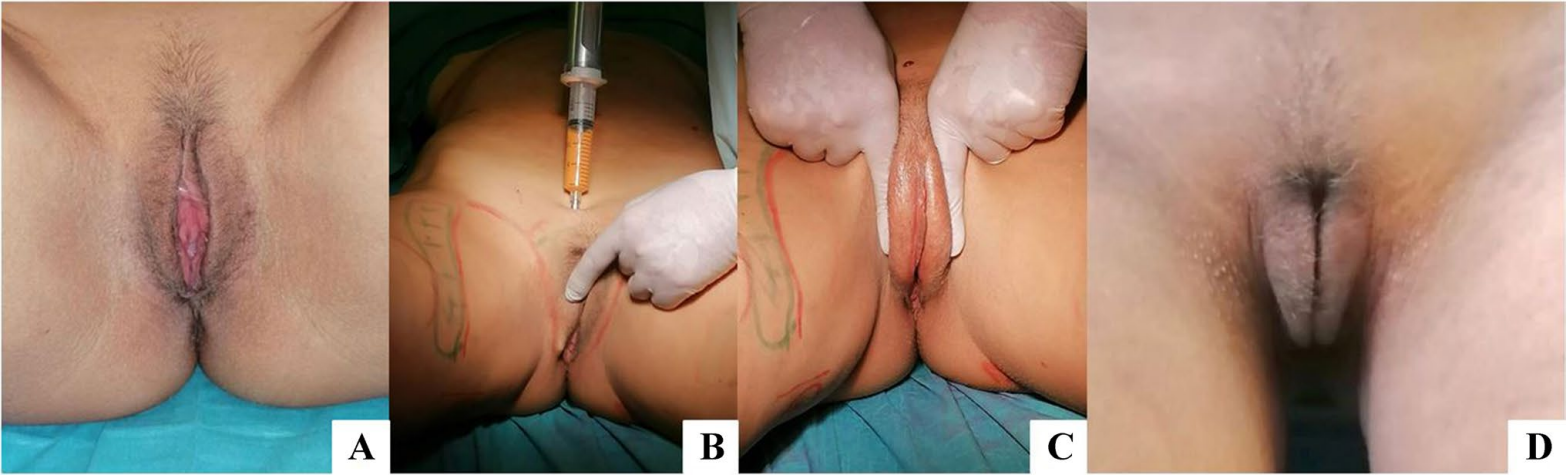
Labia majora augmentation to reduce the exposition of labia minora. **A** Preoperative image showing inner labia exposition, **B** REMIX gyno type 1 injection, **C** biograft redistribution and assessment, **D** postoperative image at 6 months showing satisfactory labia majora shape and volume

At the end of the procedure, the BACTs can be grafted into the recipient site (Fig. 16).

**Fig. 17:**
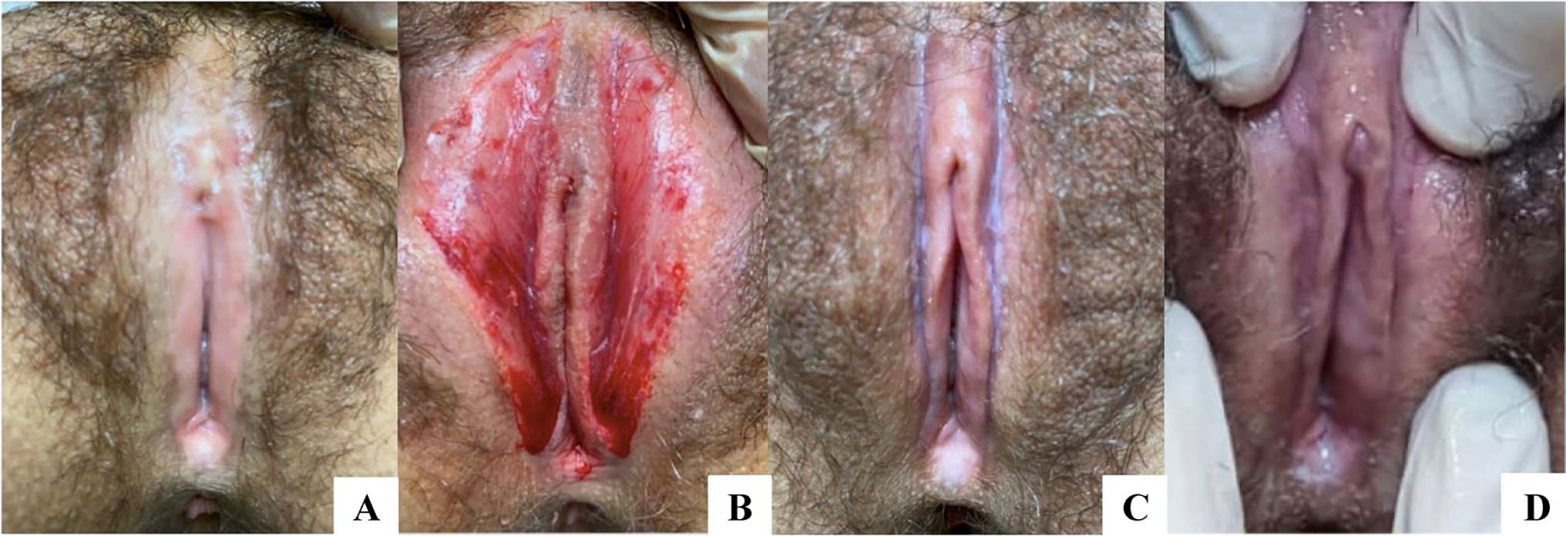
The gyno type 2-BACT components improve local regeneration of the genital area. Vulvar/vaginal repair (5/10 cc) of lichen sclerosus **A** preoperative image; **B** debridement with CO_2_ laser followed by BACTs REMIX gyno typo2, **C, D** 4 months post-op showing the total mobility of clitoral hood (courtesy of Dr. J. Elias)

On the other hand, the REMIX gyno type 2 for vulvar/vaginal repair is composed by 50% of freshly insulated SVF, 50% of PRF, 50 mg × cc of amino acids and 150 mgs × cc of vitamins and 40 mg × cc of GSH (Table 10 B). The SVF has to be enriched in a percentage of 300% and the PRF has to be spinned for 4 times before mixing the components. Finally, the injection has to be performed under direct visual control (Fig. 17).

### Pulmonary fibrosis

During the COVID-19 pandemic, we have witnessed an exponential blooming of the number of tutorials and webinars that made it possible to share ideas among scientists all over the world and at the same time showed the importance of searching and pioneering new effective and safe procedures able to reduce invasiveness and speed up recovery time, as described by Mayer and Persichetti [60].

As a matter of fact, regenerative medicine and cell therapy represent a great therapeutic option for the treatment of both acute and chronic lung diseases, such as ARDS, idiopathic pulmonary fibrosis, systemic fibrosis lung disease, and COPD. Numerous authors proposed the use of therapy based on mesenchymal stem cells in the treatment of COVID-19 pneumonia sequela [61–64]. 

Stem cells have been shown to be immune to virus infection [65]. Preclinical studies on animal models of pulmonary fibrosis and emphysema have demonstrated the role of mesenchymal stem cells harvested from bone marrow, umbilical cord, or adipose origin [66–69]. The paracrine modulatory action of MSCs on lung cells together with their ability to differentiate favoring the repair of tissue lesions are the mechanisms of action limiting scarring and promoting regeneration.

The endobronchial administration of ADSC precursors was demonstrated safe in a previous study [70]. The active role of the patient could support therapy effect during the three phases of breathing, inspiration, holding, and expiration.

Prospective, nonrandomized, uncontrolled clinical studies will allow the confirmation of the safety and efficacy of the endobronchial administration of BACTs. The creation of specific REMIX including reactive oxygen species (ROS) scavengers would allow the reduction of pulmonary damage.

## Ongoing researches and new perspectives

As mentioned before, there are unfortunately many factors, which can heavily interfere with regeneration and cell integration into the recipient site. In addition to the above-described strategies to improve transplanted cells survival, the authors are actively working on new lines of research in order to improve the regenerative response. The redirection of cytokine pools into a probiotic pathway, the stimulation of muse population cells through a specific donor site preparation and delayed harvesting phase, photobiomodulation and exertional gene expression are important pillars of our new strategies (Table 11).

**Table 11:** Ongoing research on new strategies to improve transplanted cell survival

Strategies	Effect
Jakinhibits	Cytokine modulation
Donor site preparation	Cell/ECM enrichment
Delayed harvesting	Muse stimulation
Muse cells	Cellular boost
Photobiomodulation	Cellularity and replication
Exosomes	Paracrine factors

### Cytokine modulation

As already analyzed at the beginning of this article, one of the most important causes that can sustain an increase in the apoptotic death of transplanted cells and consequently significantly compromise the regenerative activity is represented by the important inflammatory response at the recipient site level after grafting. The inflammatory response is mainly determined by the activation of a complex cytokine pool due to the direct surgical trauma and the local reaction sustained by the body's defenses [71]. It is therefore important to be able to limit this inflammatory response by modulating cytokine activity and containing the antagonistic action against the bioactive therapies, meanwhile redirecting the inflammatory response toward a probiotic pathway, which does not interfere with cellular survival mechanisms.

Recent studies have highlighted the fact that local modulation of cytokines (e.g., IL-1, IL-6, TGF alpha) significantly improves transplanted cell intake and proliferation. As previously explained, cellular and blood components contained in BACTs are in many ways concurring in reducing inflammatory response by cytokine modulation but a greater action can be obtained by a focused inhibiting action of Janus kinase (JAKs) activity. JAKs are specific signal transducers and activators of transcription (STATs) associated with one of the most important pathways in which cytokines integrate their functions: the JAK/STAT pathway. After the binding of their respective effector molecules (cytokines, IFNs, GF, and many other hormone receptors) to type I and II receptors, JAKs become activated and they mediate the signal transduction to the nucleus, resulting in synthesis of bioactive compounds interfering with cell metabolism and functions. All these mechanisms are well known to play a very important role in many autoimmune diseases and since long time now are one of the hot topics in modern rheumatologic strategies [72]. Recently, a new family of oral drugs named JAK kinase inhibitors (Jakinhibits) has been developed. Jakinhibits are a group of small molecules strictly involved in inflammatory diseases currently marketed for rheumatoid arthritis (RA) and psoriatic arthritis. The efficacy and safety of JAK inhibitors has been extensively proved for many immune-mediated clinical conditions and as per today these drugs are undergoing phase 2 and 3 of several clinical trials for many other inflammatory diseases such as alopecia aerata. The use of tofacitinib (Xeljanz) with a posology of 5 to 10 mg per day to treat severe rheumatoid arthritis showed great efficacy in controlling symptoms and evolution and is well tolerated by the patients even in long-term treatments [73].

In close collaboration with the rheumatologists, the authors carried on several clinical trials for evaluating the possibility of improving outcomes of advanced regenerative procedures using lower doses for a limited time. For improving outcomes in regenerative surgery, a protocol using 2.5 mg per day 1 week before the grafting and 4 weeks after the surgery showed great efficacy with very limited side effects in controlling inflammatory response. Main limits of this novel strategy are the relevant cost and the “off label” use but authors believe that is worth going through with further clinical trials especially for complicated and multi stage regenerative procedures such as diabetic foot and burns.

### Preconditioning procedures: delayed harvesting

This is a novel strategy to selectively increase in vivo the nucleated cell number and induce a selective cell redirecting through a preparation phase of the donor area. By producing repeated mechanical, chemical, or thermal stresses, it is possible to stimulate pericyte freeing and activation, inducing repairing processes and a great proliferation concentration of very powerful MSCs called MUSE cells.

This process continues in the injured tissues with infiltration of circulating cells and the migration of cells from adjacent areas, such as fibroblasts. The latter, in synergism with the other local cells previously activated, will be the protagonists of fibroplasia and depositors of ECM, angiogenesis, and wound healing triggering the activation of their reparative potential which will increase once transplanted in the recipient site.

Under ideal conditions, the possibility to isolate, expand, and cultivate in vitro MUSE cells, even if not so easy to be done, could allow to selectively work just on this specific cell type able to ensure a higher regenerative potential. However, as mentioned above, the current regulatory frame does not allow to perform HGM procedures, hence, the authors developed a novel strategy to selectively increase in vivo the concentration of MUSE cells through a preparation phase of the donor area. This could represent a fundamental aspect of the new paradigm to improve nucleated cells and precursor rate in harvested SVF. Tissue damage of any kind (physical, chemical, or biological) triggers an immediate series of signaling events initiated by chemical structures that are made by ruptured cells (portions of the cell membrane and organelles), fragments of inert elements of tissues (collagen, elastin, laminin, fibronectin, and other ECM components), and the action performed by inflammatory mediators mainly released from platelets and mast cells or neosynthesized by platelet-activating factors.

Migration of lymphocytic cells and connective tissue formation, indicating a healing process has been observed after closed lipoclasia [74]. This is because intracellular lysosomal rupture induces the release of vasoactive kinins causing a localized mild inflammatory reaction. Lymphocytes are the most abundant leukocyte subsystem found in the conditioned site and, as well known, they not only act as immune effectors, but also producing GFs responsible for the rebuilding of regional cellularity and restoring their homeostasis. This process continues in the injured tissues with infiltration of circulating cells and the migration of cells from adjacent areas, such as fibroblasts. The latter, in synergism with the other local cells previously activated, will be the protagonists of fibroplasia and depositors of ECM, angiogenesis, and wound healing triggering the activation of their reparative potential which will increase once transplanted in the recipient site.

**Fig. 18:**
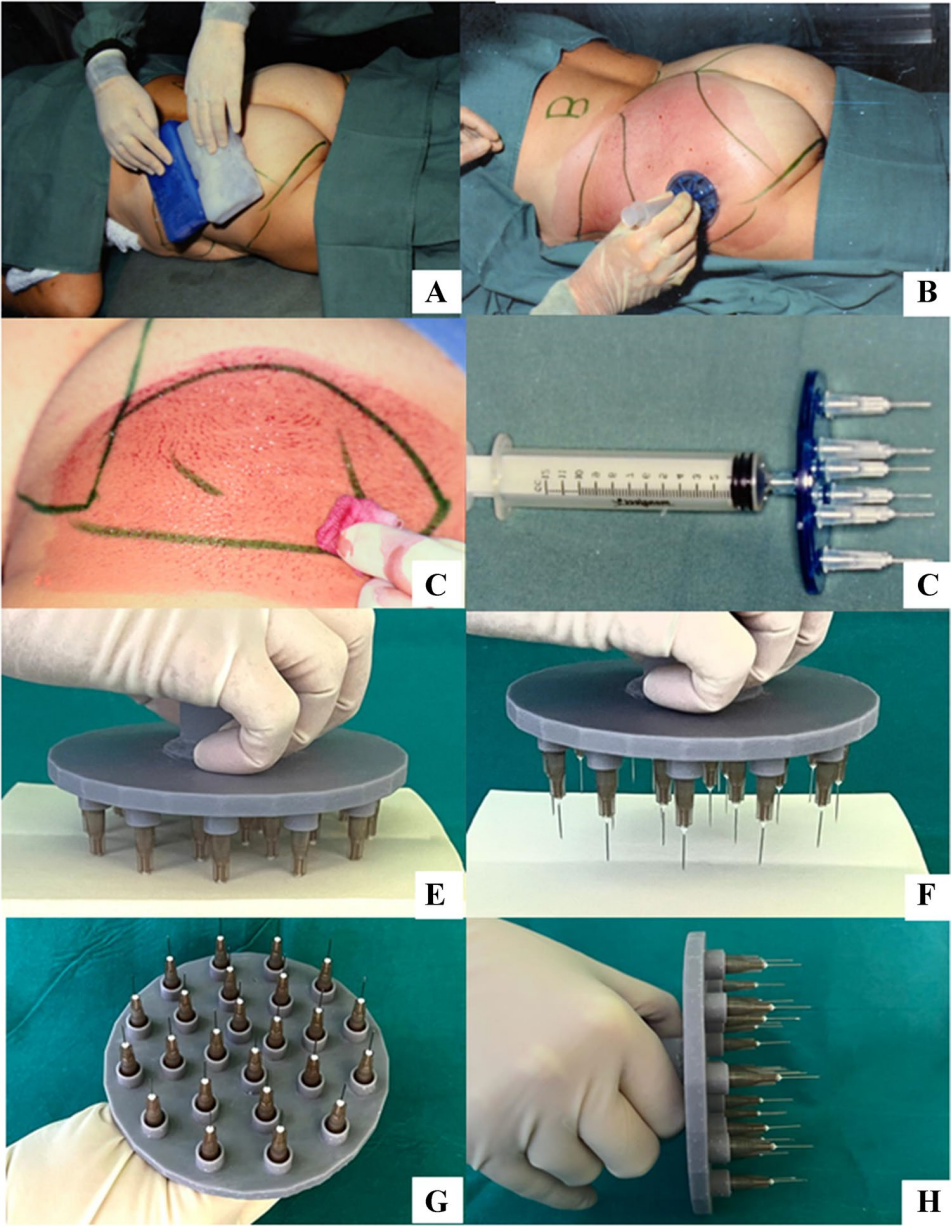
**A** Cryoanesthesia; **B** biostimulation; **C** hundreds of punctures in the first 2 cm of adipose tissue using a needling plate holding seven 15-mm 27-g needles **D** old instrument. **E**, **F**, **G**, **H** The authors developed a new biostimulator to hold up to 25 needles for speeding up this technical step

In practice, by producing repeated mechanical, chemical, or thermal stresses, it is possible to stimulate pericyte freeing and activation, inducing repairing processes, increasing the nucleated cell number and a selective cell redirecting. A total of 5/7 weeks before the scheduled regenerative procedure a localized trauma is induced, after cryoanesthesia, in the donor areas, producing hundreds of punctures in the first 2 cm of adipose tissue using a needling plate holding seven 13- to 15-mm 27-g needles (Fig. 18).

This induced but controlled trauma, even if can be at the origin of apoptotic death of some fragile precursors, is unleashing a strong reparative reaction freeing a great number of pericytes meanwhile stimulating the selective proliferation of more resistant cells line: the MUSE cells. Most lymphoid cells found 3 days post-procedure were lymphocytes with few neutrophils and macrophages. The number of neutrophils is strictly related to the level of asepsis during the procedure, which highlights the importance of the procedure being performed by a trained medical professional only in highly equipped surgical facilities.

The analyses carried out on tissue samples harvested at 3, 4, and 5 weeks clearly shown that through delayed harvesting, due to the higher population of fibroblasts, after only 3 to 4 weeks from the pretreatment collagen types II and III, elastin and laminin content is increased.

As a direct result of the richer content of ECM main components, all the MSC precursors remain protected by a denser matrix stimulating niches’ activity and homeostasis and after 5 to 6 weeks the local MUSE cells concentration can reach up to 5%. In conclusion donor area preconditioning has shown great capability to increase ECM concentration and local population of MUSE cells, the increased concentration of MUSE cells is highly enriching the harvested SVF of very active and stress-resistant cellular population, this feature being important especially when the volume of the recipient site is very small such as ATM joints or vocal cords [75–77].

### MUSE cells

The multilineage-differentiating stress-enduring (MUSE) cells are an important component of the AT, newly discovered in 2010 by the scientists of the Tohoku University of Japan [78]. Being classified as endogenous noncancerous pluripotent stem cells, they represent around 1–3% of total l MSC population [79]. MUSE cells present highly preserved cellular mechanisms and are stress tolerant, with a significant capacity to self-renew and to differentiate into cells of all three mesenchymal germ layers.

In contrast to embryonic and induced pluripotent stem cells (IPSCs), they exhibit a normal karyotype, the have a low telomerase activity and they do not undergo tumorigenesis one implanted in the SCID mice. Furthermore, the senescence level and apoptosis rates are markedly lower compared with other cells. Because of all these capacities, MUSE cells home into damaged tissue in a very effective way than any other population of MSCs.

MUSE cells can be identified from the isolated mesenchymal stem cells population positive for CD90, CD73, CD105, CD44, CD29, and negative for CD45 and CD34, isolating cells expressing simultaneously CD105 and SEEA3 as described by Conti et al. applications [80].

In humans, a high concentration of MUSE cells has been found in the anterior region of the thighs [81].

**Fig. 19:**
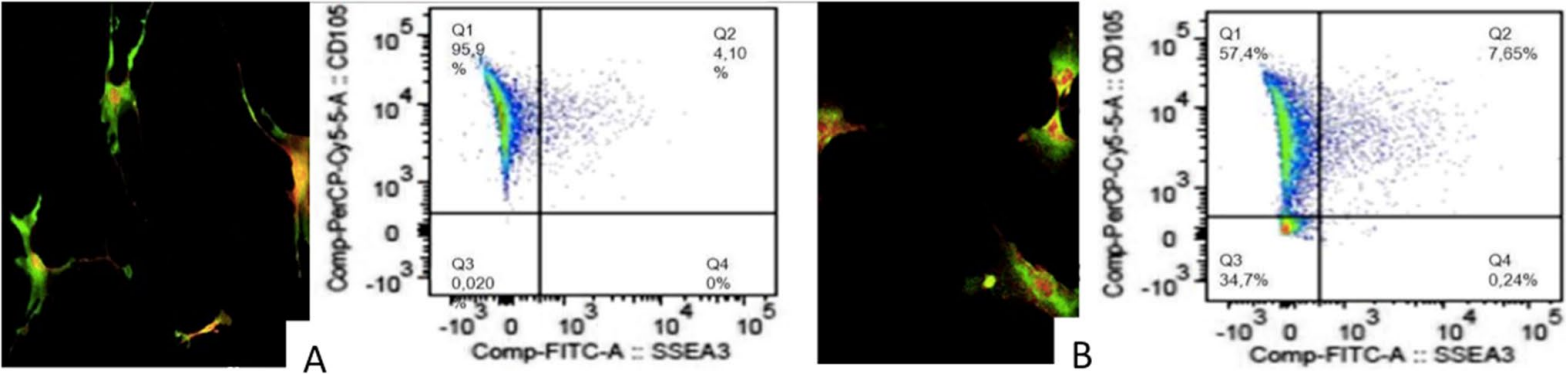
Concentration of MUSE cells in the gluteal fat pad. **A** Before biostimulation of gluteal fat pad 4.10% of MUSE cells. **B** After biostimulation, the concentration of MUSE cells changed to 7.65% (courtesy of A. Sbarbati and G. Conte)

More recent studies showed that the concentration of muse cells varies significantly according to the different anatomical districts of the body. In the abdominal area, for example, the number of muse is very limited (3/5%) while in anterior face of the tights and in the pretrochanteric region their number slightly increases up to 6/7%). In other anatomical areas subjected to constant mechanical trauma, such as the lower medial part of the buttocks destined to support the body weight in sitting position, reveals much higher muse concentrations (up to 50%). Unfortunately, the removal of adipose tissue in that area is contraindicated because it is essential to keep a thickness of the adipose panniculus sufficient to ensure a cushioning effect in a sitting position to avoid skin suffering and even appearance of pressure ulcers. The most amazing example of the strict correlation between mechanic trauma and number of music cells can be found at the level of Bichat bubble where the percentage of muse is very high (up to 90%) but unfortunately not so suitable for standardized clinical [80] (Fig. 19).

These findings are at the origin of the rationale of biostimulation of the anatomical areas most suitable for sampling, such as the pretrochanteric area and the supero-external area of the buttocks.

The possibility of increasing the number of MUSE cells in the cellular components of BACTs is very appealing due to their higher resistance to metabolic and mechanical stress. However, the areas rich in MUSE cells offer limited possibility of AT harvesting due to the risk of complication in the femoral fat pad and limited amount of AT available in the Bichat’s fat pad.

### Exosomes

Exosomes are small spherical membrane vesicles originating from the late endosomal membrane secreted by living cells. They were initially considered discarded cell remnant containing proteins, nucleic acids, microRNA, mRNA, DNA, and other molecules, while they are known to act as important mediator of paracrine cell communication [82].

When exosomes are absorbed by specific target cells, the exosomal contents, especially miRNAs, will mediate numerous biological processes. In addition to a role in cancer progression and immunoregulation. They are involved in mechanisms of tissue repair and regeneration.

The main evidence supporting this originates from investigations focusing on mesenchymal stem cell (MSC) transplantation for tissue regeneration. Currently, it is believed that MSCs achieve a therapeutic effect in vivo mainly through paracrine signaling. They can release biologically active molecules that affect the proliferation, migration, and survival of the neighboring cells.

Numerous preclinical studies have confirmed that MSC exosomes play a key role in tissue regeneration and repair, particularly in cutaneous wound healing. MSC exosomes participate in each phase of the cutaneous wound healing processes by delivering various molecules, such as trophic factors, functional proteins, and RNAs, including mRNA and miRNAs [83].

Exosomes are the main bioactive vesicles responsible for the paracrine effects of MSCs; they in fact regulate many physiological and pathological processes by affecting the survival, proliferation, migration, and gene expression of recipient cells and by programing targeted cell behaviors.

On this evidence, it could be possible to adopt a cell-free therapy utilizing paracrine factors, such as exosomes, to promote tissue repair and regeneration, which would avoid the risks associated with direct stem cell transplantation, such as teratomas, immune rejection, and the reduced regenerative capacity of engrafted cells.

### Photobiomodulation

Photobiomodulation (PBM) nonionizing photonic energy induces local photochemical changes. From the cellular and molecular point of view, visible red and near infrared light energy (400 to 480 nm) are mainly absorbed by mitochondria. The mitochondrial enzyme cytochrome c oxidase, acting as a chromophore, accepts the photonic energy deriving from PBM, causing ATP, NO, and mild oxidant production and the activation of cellular repair and healing mechanisms, with significant impact on cellularity and differentiation [84].

Similar mechanical stimulations have been shown to trigger cell progenitors from quiescent “sleeping precursors” within the AT niches to the activated status. After harvesting, centrifuging, and modulating the lipoaspirated fat sample treated with a 430-nm LED photobiomodulation (PBM) (Adi-Light 1, Adi-Stem Ltd., Hong Kong) showed an activation of the quiescent adipose stem cells, which became fully functional and can immediately return through an IV injection to the patient [85].

Considering the current importance of photobiomodulation in the course of evolution, the authors expect a fundamental role even within the different molecular mechanisms involved in our laboratory and clinical experiments. The rational and the mechanism of this claimed “photobiostimulation” should be better explained. In fact, in support of the alleged photo biostimulating capabilities, it would be desirable to perform comparative analysis including not only the phenotypic expression of CD 105 but extended to other criteria such as plastic adherence, expression of CD73, CD90, and CD105, and lack of expression of CD11b, CD14, CD19, CD45, and HLA-DR (as per IFATS/ISCT guidelines [86]).

### Conclusions

The most important limit of current regenerative approaches is that scientists and medical professionals have the unavoidable obligation to comply with rules, laws, and regulations becoming every day more severe and restrictive. Hence, these procedures cannot exploit the real possible benefits of advanced and powerful tools such as tissue engineering and of pro-survival gene transfer.

Nonetheless, surgeons and scientists involved in translational technology in regenerative therapies should focus on affordable strategies, allowing their diffuse application in all the settings of treatment, avoiding the utilization of very expensive technologies with minimal improvement of nucleated cell isolation.

Moreover, we do not already have the possibility to quantify the stemness of our grafts because we cannot perform HGM procedures such as mesenchymal stem cell cultivation and expansion. For all these reasons, we do not have the possibility to deeply analyze the in vivo proliferation and differentiation levels of our regenerative mixtures.

Even if cell sorting and immunophenotyping evaluations cannot be performed for each single patient worldwide, dependable and reliable technologies should be offered to our patients. Minimal values of the most important parameters of regenerative strategies, such as nucleated cells concentration, viability, and percentage of mesenchymal progenitors, type and concentration of GFs, should be defined by the international community.

The fresh regenerative mixture induces the recipient site to act as a real in vivo bioreactor. Future preclinical and clinical studies are needed in order to define the best recruiting activity, cell sorting, differentiation, and cellularity to the recipient sites. In addition, further study will aid in defining the best REMIX (REgenerative MIXture) for different clinical needs and anatomical districts.

In this manuscript, authors presented an overview about BACTs and the rationale behind our newly developed regenerative technology. This paper confirms once again the role of translational medicine in tissue engineering and regenerative medicine. Sharing this new approach to regenerative medicine will improve the efficacy of regenerative medicine procedures respecting regulatory frames related to cell manipulation limits for the safety of our patients.

In addition to the above-described strategies to improve transplanted cell survival, the authors are working on new lines of research in order to improve the regenerative response such as the redirection of cytokine pools into a probiotic pathway and the stimulation of MUSE cells through a specific donor site preparation and a delayed harvesting phase. Even photobiomodulation and exertional gene expression are important pillars of our new strategies.

The data merging from our experience in the last 4 years are extremely promising but it’s now mandatory to further confirm the solid rationale behind this intuition providing additional data and further elucidate the therapeutic value of using this new regenerative strategy to consolidate outcomes and results.

It is easy to predict that shared guidelines should be established, in close collaboration with many other specialists, for standardizing typology and proportion of the different regenerative components to be adapted to the different clinical and anatomical situations, depending on whether the need to stimulate the regenerative processes or to modulate the inflammatory response is prevalent.
